# Immunogenicity of the RTS,S/AS01 malaria vaccine and implications for duration of vaccine efficacy: secondary analysis of data from a phase 3 randomised controlled trial

**DOI:** 10.1016/S1473-3099(15)00239-X

**Published:** 2015-12

**Authors:** Michael T White, Robert Verity, Jamie T Griffin, Kwaku Poku Asante, Seth Owusu-Agyei, Brian Greenwood, Chris Drakeley, Samwel Gesase, John Lusingu, Daniel Ansong, Samuel Adjei, Tsiri Agbenyega, Bernhards Ogutu, Lucas Otieno, Walter Otieno, Selidji T Agnandji, Bertrand Lell, Peter Kremsner, Irving Hoffman, Francis Martinson, Portia Kamthunzu, Halidou Tinto, Innocent Valea, Hermann Sorgho, Martina Oneko, Kephas Otieno, Mary J Hamel, Nahya Salim, Ali Mtoro, Salim Abdulla, Pedro Aide, Jahit Sacarlal, John J Aponte, Patricia Njuguna, Kevin Marsh, Philip Bejon, Eleanor M Riley, Azra C Ghani

**Affiliations:** a MRC Centre for Outbreak Analysis and Modelling, Department of Infectious Disease Epidemiology, Imperial College London, London, UK; b Kintampo Health Research Centre, Kintampo, Ghana; c London School of Hygiene & Tropical Medicine, London, UK; d Tanzania National Institute for Medical Research, Tanzania; e University of Copenhagen, Copenhagen, Denmark; f School of Medical Science, Kwame Nkrumah University of Science and Technology, Kumasi, Ghana; g Agogo Presbyterian Hospital, Agogo, Ghana; h KEMRI-Walter Reed Project, Kombewa, Kenya; i Centre de Recherches Médicales de Lambaréné, Lambaréné, Gabon; j Institut für Tropenmedizin, Universitätsklinikum Tübingen, Tübingen, Germany; k German Center for Infection Research, Germany; l Department of Medicine, University of North Carolina, Chapel Hill, NC, USA; m University of North Carolina Project-Malawi, Lilongwe, Malawi; n Institut de Recherche en Sciences de la Sante, Nanoro, Burkina Faso; o KEMRI/CDC Public Health and Research Collaboration, Kisumu, Kenya; p Malaria Branch, Division of Parasitic Diseases and Malaria, US Centers for Disease Control and Prevention, Atlanta, GA, USA; q Bagamoyo Research and Training Centre, Ifakara Health Institute, Bagamoyo, Tanzania; r Centro de Investigação em Saúde de Manhiça, Manhiça, Mozambique; s Faculdade de Medicina, Universidade Eduardo Mondlane, Maputo, Mozambique; t ISGlobal, Barcelona Centre for International Health Research, Hospital Clinic - Universitat de Barcelona, Barcelona, Spain; u KEMRI Wellcome Trust Research Programme, Kilifi, Kenya; v Nuffield Department of Medicine, University of Oxford, Oxford, UK; w African Academy of Sciences, Nairobi, Kenya

## Abstract

**Background:**

The RTS,S/AS01 malaria vaccine targets the circumsporozoite protein, inducing antibodies associated with the prevention of *Plasmodium falciparum* infection. We assessed the association between anti-circumsporozoite antibody titres and the magnitude and duration of vaccine efficacy using data from a phase 3 trial done between 2009 and 2014.

**Methods:**

Using data from 8922 African children aged 5–17 months and 6537 African infants aged 6–12 weeks at first vaccination, we analysed the determinants of immunogenicity after RTS,S/AS01 vaccination with or without a booster dose. We assessed the association between the incidence of clinical malaria and anti-circumsporozoite antibody titres using a model of anti-circumsporozoite antibody dynamics and the natural acquisition of protective immunity over time.

**Findings:**

RTS,S/AS01-induced anti-circumsporozoite antibody titres were greater in children aged 5–17 months than in those aged 6–12 weeks. Pre-vaccination anti-circumsporozoite titres were associated with lower immunogenicity in children aged 6–12 weeks and higher immunogenicity in those aged 5–17 months. The immunogenicity of the booster dose was strongly associated with immunogenicity after primary vaccination. Anti-circumsporozoite titres wane according to a biphasic exponential distribution. In participants aged 5–17 months, the half-life of the short-lived component of the antibody response was 45 days (95% credible interval 42–48) and that of the long-lived component was 591 days (557–632). After primary vaccination 12% (11–13) of the response was estimated to be long-lived, rising to 30% (28–32%) after a booster dose. An anti-circumsporozoite antibody titre of 121 EU/mL (98–153) was estimated to prevent 50% of infections. Waning anti-circumsporozoite antibody titres predict the duration of efficacy against clinical malaria across different age categories and transmission intensities, and efficacy wanes more rapidly at higher transmission intensity.

**Interpretation:**

Anti-circumsporozoite antibody titres are a surrogate of protection for the magnitude and duration of RTS,S/AS01 efficacy, with or without a booster dose, providing a valuable surrogate of effectiveness for new RTS,S formulations in the age groups considered.

**Funding:**

UK Medical Research Council.

## Introduction

Malaria imposes an enormous burden on public health, causing an estimated 584 000 deaths worldwide in 2013, with most attributable to *Plasmodium falciparum* in African children.^1^ An effective malaria vaccine would help to protect this vulnerable population. The RTS,S/AS01 candidate vaccine for preventing *P falciparum* malaria was assessed in a phase 3 trial done between 2009 and 2014, in 11 sites in sub-Saharan Africa.^2^, ^3^ 8922 children aged 5–17 months and 6537 infants aged 6–12 weeks were randomly assigned to receive either three doses of RTS,S/AS01 once per month for 3 months and a booster dose at 20 months (R3R group); three doses of RTS,S/AS01 and a dose of comparator vaccine at 20 months (R3C); or three doses of a comparator vaccine once per month for 3 months and a booster dose at 20 months (C3C). The median time until the end of the study was 48 months after the first dose for children and 38 months for infants. Over the entire duration of the trial, vaccine efficacy against clinical malaria in children was 28% (95% CI 23–33) in the R3C group and 36% (32–41) in the R3R group. Efficacy was lower in infants: 18% (12–24) in the R3C group, and 26% (20–32) in the R3R group.

RTS,S/AS01 is a recombinant protein candidate malaria vaccine that targets the *P falciparum* circumsporozoite protein. It contains part of the circumsporozoite sequence, coexpressed with hepatitis B surface antigen, inducing anti-circumsporozoite antibodies and circumsporozoite-specific CD4-positive T cells that are associated with protection from *P falciparum* infection and episodes of clinical malaria.^4^, ^5^ Anti-circumsporozoite antibody titres might also be associated with the duration of protection, with the rate at which anti-circumsporozoite antibodies wane similar to the rate of decline of efficacy.^6^, ^7^

**Research in context**

**Evidence before this study**

We searched PubMed on June 9, 2015, for studies on the association between the immunogenicity of RTS,S and efficacy using the MeSH terms “RTS,S” and (“circumsporozoite” OR “immunogenicity” OR “antibody”). We identified 115 reports. 23 were studies of the statistical association between RTS,S-induced immune responses (anti-circumsporozoite antibody titres or circumsporozoite-specific T-cell responses) and efficacy against either *Plasmodium falciparum* infection or episodes of clinical malaria, based on data from phase 2 clinical trials. Five studies measured RTS,S-induced immune responses over a period greater than 2 years, showing associations between antibody titres and protection, and decaying antibodies over time.

**Added value of this study**

This study includes data from a large phase 3 trial spanning a wide range of malaria transmission intensities. The study combines measurements of anti-circumsporozoite antibody titres over time with individual-level data for episodes of clinical malaria to provide estimates of the duration of the antibody response over time and the association between anti-circumsporozoite antibody titres and efficacy. The decay of anti-circumsporozoite antibody titres over 4 years can be described by a biphasic exponential distribution. An anti-circumsporozoite antibody titre of 121 EU/mL (95% credible interval 98–153) was estimated to prevent 50% of infections.

**Implications of all available evidence**

The RTS,S malaria vaccine provides significant efficacy against episodes of clinical malaria in different age groups across different transmission settings. This analysis shows that RTS,S/AS01-induced anti-circumsporozoite antibody titres can be used as a correlate of protection to predict vaccine efficacy over time. The estimated relationship between anti-circumsporozoite antibody titres and efficacy can be used to assess future versions of RTS,S and second generation anti-circumsporozoite vaccines.

Vaccine protection is the probability that vaccine-induced immune responses prevent infection with pre-erythrocytic stages of *P falciparum*. It is measured most directly as efficacy against infection in controlled human malaria infection trials in malaria-naive adults.^4^, ^8^ Vaccine efficacy against clinical malaria as reported in field trials^2^, ^3^ is a relative measure of the incidence of malaria in vaccinated and control cohorts, and can be affected by immune responses apart from that induced by vaccination. In the case of RTS,S/AS01, infections prevented in vaccinated individuals might reduce anti-blood-stage immunity over time in vaccinated people compared with control participants. This effect could cause efficacy against clinical malaria to wane at a faster rate than efficacy against infection, particularly in regions of high transmission.

Assessing the duration of protection following RTS,S/AS01 vaccination remains a challenge. Clinical trials have reported vaccine efficacy as fixed point estimates,^9^ or as continuously varying estimates throughout follow-up.^10^ Several statistical methods have been used to assess waning vaccine efficacy, including testing for non-parametric patterns of waning,^11^ or the incorporation of time-dependent covariates into proportional hazards models.^7^, ^12^ In this Article, we extend existing statistical methods^6^ to evaluate the vaccine's efficacy profile, defined as the initial efficacy after vaccination and the pattern of waning over time.

## Methods

### Data

RTS,S/AS01 was tested in an individually randomised controlled double-blind phase 3 trial designed to evaluate vaccine efficacy, safety, and immunogenicity.^2^, ^3^ We analysed the immunogenicity of the vaccine after primary vaccination with or without a booster dose and assessed how efficacy against clinical malaria depends on the rate of waning of vaccine-induced antibodies and transmission intensity.

We used the primary case definition of an episode of clinical malaria: illness in a child brought to a study facility with a measured temperature of 37·5°C or more, or reported fever within the past 24 h and *P falciparum* asexual parasitaemia at a density of more than 5000 parasites per μL. We focused on the per-protocol population for which follow-up begins 3 weeks after the third dose. Serological data were available for a subset of participants from each trial site. Anti-circumsporozoite and anti-hepatitis B surface antigen antibodies were measured by standardised enzyme-linked immunosorbent assays and antigens in a single laboratory.^13^ Table 1 shows malaria transmission intensity and anti-circumsporozoite antibody titres at each trial site.

### Immunogenicity

We assessed the effects of several covariates on anti-circumsporozoite antibody titres following primary vaccination with RTS,S/AS01, and following a booster dose of RTS,S/AS01. The covariates were age at vaccination, malaria transmission intensity, HIV status, and anti-circumsporozoite and anti-hepatitis B surface antigen antibody titres at screening. We analysed the data using linear regression models with trial site as a random effect to account for additional heterogeneity not captured by the fixed effects.

### Antibody dynamics

After primary vaccination with RTS,S/AS01, anti-circumsporozoite antibody titres are assumed to increase to a peak value (CS_peak_) and then wane over time (t) according to a biphasic exponential model:

$$\text{CS}(\text{t})={\text{CS}}_{\text{peak}}(\rho_{\text{peak}}e^{-r_{s}^{t}}+(1-\rho_{\text{peak}})e^{-r_{l}^{t}})$$

where r_s_=log_e_(2)/d_s_ and r_l_=log_e_(2)/d_l_ are the decay rates of the short-lived and long-lived components of the antibody response, and ρ_peak_ is the proportion of the antibody response that is short-lived. After a booster dose at time t_boost_, antibody titres increase to CS_boost_. We assumed that the rates of decay of the short-lived and long-lived components of the antibody response remain the same, but that the proportion of the response that is short-lived (ρ_boost_) might change. For t>t_boost_ the antibody dynamics can be described as follows:

$$\text{CS}(\text{t})={\text{CS}}_{\text{boost}}(\rho_{\text{boost}}e^{-r_{s}(t-t_{\text{boost}})}+(1-\rho_{\text{boost}})e^{-r_{l}(t-t_{\text{boost}})})$$

### Association between anti-circumsporozoite antibodies and clinical malaria

The pattern of waning of antibody titres can be used to estimate the change in vaccine efficacy over time. For estimated anti-circumsporozoite antibody titres, vaccine efficacy against infection can be estimated with a dose–response curve defined as follows:

$$V(t)=V_{\max}\left(1-\frac{1}{1+{\left(\frac{CS(t)}{\beta }\right)}^{\alpha }}\right)$$

where V_max_, α, and β are parameters to be estimated. RTS,S/AS01-induced anti-circumsporozoite antibodies prevent episodes of clinical malaria by reducing or preventing pre-erythrocytic *P falciparum* infection. To investigate the association between anti-circumsporozoite antibodies and clinical malaria, we used this equation to estimate the probability of infection. To account for the probability that blood-stage infection progresses to a detected episode of clinical malaria, we used a model for the age-dependent and exposure-dependent acquisition of anti-blood-stage immunity.^14^ This model accounted for a trial site's transmission intensity, age, heterogeneity and seasonality in exposure, and bednet use. The model predicts that the prevention of infections by RTS,S/AS01 reduces naturally acquired anti-blood-stage immunity in the vaccine cohort compared with the control cohort. This effect results in a higher probability of blood-stage infections progressing to symptomatic episodes of clinical malaria in the vaccine cohort.^14^

### Statistical analysis

We fitted the antibody dynamics model to longitudinal data on anti-circumsporozoite antibody titres in a Bayesian framework using Markov chain Monte Carlo methods with mixed effects to capture between-individual variation. The parameters describing the association between anti-circumsporozoite antibodies and efficacy against infection in the equation for the dose–response curve were estimated by fitting to individual-level data on times of episodes of symptomatic malaria (primary case definition, per-protocol population) with survival analysis methods in a Bayesian Markov chain Monte Carlo framework. Best fit parameters were taken to be the medians of the estimated posterior distributions. Parameters are presented with 95% credible intervals (CrI; appendix).

### Role of the funding source

The sponsors had no role in the design of this secondary analysis, in doing the analysis, interpreting the data, or writing this report. The corresponding author had full access to all the data in the study and had final responsibility for the decision to submit for publication.

## Results

Table 2 shows the dependence of anti-circumsporozoite antibody titre after primary vaccination or a booster dose on covariates. RTS,S/AS01 was more immunogenic in children aged 5–17 months than in those aged 6–12 weeks. Being HIV positive was associated with reduced immunogenicity. Within the 5–17 month age category, younger children had higher anti-circumsporozoite antibody titres after vaccination. In children aged 6–12 weeks, high baseline anti-circumsporozoite antibody titres were associated with low anti-circumsporozoite antibody titres after vaccination, suggesting that maternal antibodies or fetal exposure to malaria parasites might inhibit immunogenicity.^6^, ^15^ We recorded no significant associations between baseline anti-hepatitis B surface antigen antibodies and immunogenicity (appendix).

The booster dose was more immunogenic in children aged 5–17 months than those aged 6–12 weeks. There were no significant associations between age and booster dose immunogenicity. The most significant predictor of anti-circumsporozoite antibody titre after the booster dose was anti-circumsporozoite antibody titre after primary vaccination. This finding might be a result of the fact that individuals with higher capacity to respond to vaccination have higher responses at both timepoints, but might also be because the residual effects of priming by the primary vaccination leads to more effective boosting.

**Figure 1:**
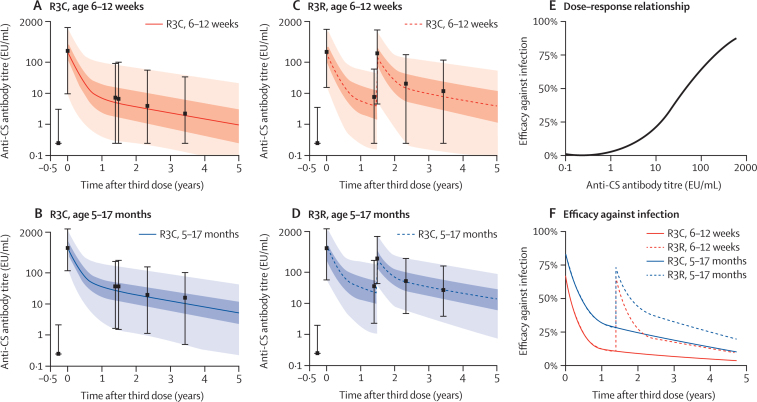
Anti-circumsporozoite antibody dynamics and association with efficacy against infection (A–D) Anti-circumsporozoite antibody dynamics after a primary schedule of RTS,S/AS01 with or without booster. The black bars denote the median and 95% ranges (2·5–97·5 percentile). The solid and dashed curves denote the median of the model predicted antibody titres. The dark and light shaded regions represent 50% and 95% of the model predicted variation in antibody titres. (E) Estimated dose–response relationship for the association between anti-CS antibody titre and efficacy against infection. (F) Estimated vaccine efficacy profile for infection based on waning antibody titres. CS=circumsporozoite. R3C=three doses of RTS,S/AS01 and a booster with a comparator vaccine. R3R=three doses of RTS,S/AS01 and a booster with RTS,S/AS01.

The dynamics of anti-circumsporozoite antibody titres after vaccination with or without a booster dose of RTS,S/AS01 are well described by a biphasic exponential model (figure 1A–D). The short-lived component of the antibody response wanes rapidly within the first 6 months, with the long-lived component waning over the next 4 years (table 3). The waning of antibody titres after the booster dose follows a similar pattern to that after the primary schedule; however, the proportion of the response that is long-lived was estimated to increase. In children aged 5–17 months, 12% (95% CrI 11–13) of the response is estimated to be long-lived after primary vaccination, increasing to 30% (28–32) after the booster dose. In children aged 6–12 weeks, 7% (6–8) of the response was estimated to be long-lived after primary vaccination, increasing to 21% (18–23) after the booster dose.

Figure 1E shows the estimated dose–response relationship between anti-circumsporozoite antibody titres and efficacy against infection. Efficacy against infection was predicted to increase smoothly with antibody titre—we found no threshold for protection.^16^ Our model predicts that an anti-circumsporozoite antibody titre of 121 EU/mL (95% CrI 98–153) prevents 50% of infections. The vaccine efficacy profile against infection can be obtained by combining the antibody dynamics and the dose–response relationship. A biphasic pattern of waning efficacy was present mirroring the pattern of decay of anti-circumsporozoite antibodies (figure 1F). In children aged 5–17 months, efficacy against infection is estimated to begin at 74% (95% range [2·5–97·5 percentile] 46–85) and wanes to 28% (5–59) at 12 months, and 9% (1–32) after 5 years. A booster dose at 18 months increases efficacy to 59% (95% range 17–80), resulting in 17% (2–43) efficacy at 5 years. In children aged 6–12 weeks, efficacy against infection was estimated to begin at 63% (95% range 18–82) and waned to 11% (1–42) at 12 months, and to 3% (1–19) after 5 years. A booster dose at 18 months increases efficacy to 58% (95% range 8–80), resulting in 8% (1–35) efficacy at 5 years.

**Figure 2:**
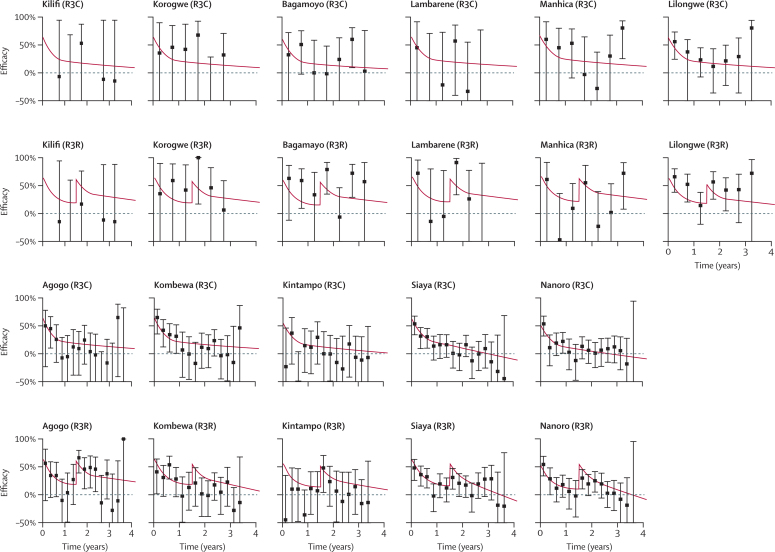
Vaccine efficacy profile for clinical malaria in children aged 6–12 weeks Data are point estimates of efficacy with 95% CIs, presented in 6 month and 3 month windows in low and high transmission sites, respectively. Kilifi, Korogwe, Bagamoyo, Lambarene, Manhica, and Lilongwe are low transmission sites. Agogo, Kombewa, Kintampo, Siaya, and Nanoro are high transmission sites. Cases of malaria are based on the primary case definition in the per-protocol population from 2·5 months to study end. The posterior median estimates of efficacy against clinical malaria predicted by the antibody dynamics model are presented in red. R3C=three doses of RTS,S/AS01 and a booster with a comparator vaccine. R3R=three doses of RTS,S/AS01 and a booster with RTS,S/AS01.

**Figure 3:**
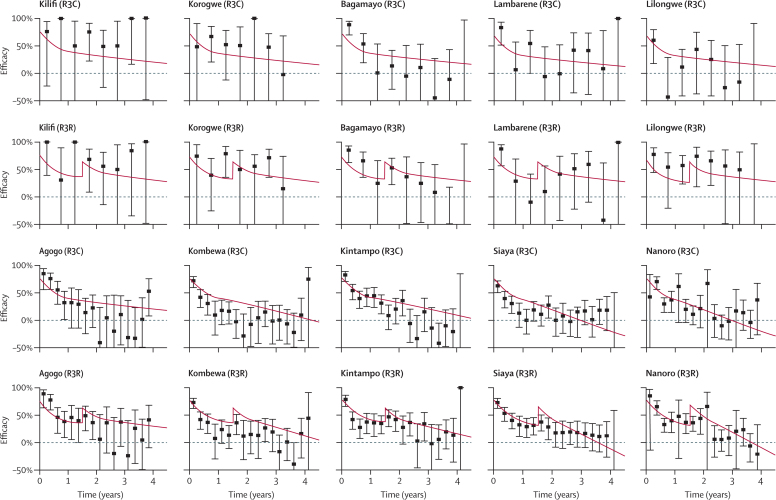
Vaccine efficacy profile for clinical malaria in children aged 5–17 months Data are point estimates of efficacy with 95% CIs, presented in 6 month and 3 month windows in low and high transmission sites, respectively. Kilifi, Korogwe, Bagamoyo, Lambarene, and Lilongwe are low transmission sites. Agogo, Kombewa, Kintampo, Siaya, and Nanoro are high transmission sites. There were no data for infants aged 5–17 months Manhica in the per-protocol cohort. Cases of malaria are based on the primary case definition in the per-protocol population from 2·5 months to study end. The posterior median estimates of efficacy against clinical malaria predicted by the antibody dynamics model are presented in red. R3C=three doses of RTS,S/AS01 and a booster with a comparator vaccine. R3R=three doses of RTS,S/AS01 and a booster with RTS,S/AS01.

For participants from all cohorts in all sites and both age categories, the antibody dynamics model predicted a vaccine efficacy profile for infection that depends on anti-circumsporozoite antibodies according to the dose–response relationship in figure 1E). The vaccine efficacy profile for clinical malaria will depend on transmission intensity and seasonality at each trial site (Figure 2, Figure 3). The difference between efficacy against infection and efficacy against clinical malaria is caused by the higher levels of naturally acquired immunity in the control group than in the vaccine group, and is predicted to be greater in sites with higher transmission intensity. For example, in Kilifi (where transmission is low), efficacy against clinical malaria is predicted to be roughly equal to efficacy against infection (Figure 2, Figure 3). By contrast, in Nanoro (where transmission is high), efficacy against clinical malaria is predicted to be substantially lower than efficacy against infection.

## Discussion

In the identification of correlates of protection against *P falciparum* infection, different trial designs provide different categories of evidence. Controlled human malaria infection trials provide the most direct evidence because mosquito infection can be controlled and immune responses measured on the day of challenge.^8^, ^17^ Field trials with an endpoint of naturally acquired *P falciparum* infection provide valuable evidence but are limited by heterogeneity in exposure.^18^ Field trials with clinical malaria as an endpoint also provide valuable evidence but are complicated by the effect of vaccination on the acquisition of clinical immunity.^10^ RTS,S-induced anti-circumsporozoite antibody titres are associated with protection in each of these types of trial.^4^, ^5^, ^6^, ^19^ Here, we provide further validation that anti-circumsporozoite antibodies are a surrogate of protection against clinical malaria using data from a phase 3 trial. For both primary vaccination and booster dose of RTS,S/AS01, anti-circumsporozoite antibodies predict efficacy against clinical malaria in both age categories across all 11 sites over the duration of the trial, thus satisfying the Prentice criteria (appendix).^20^ In the terminology proposed by Qin and colleagues,^21^ anti-circumsporozoite antibodies are a level 2 surrogate of protection because vaccine efficacy is predicted across different settings and age groups. The model was fitted to data from the subset of participants in the serology cohort, and was predictive of efficacy in the full per-protocol population.

The association between RTS,S/AS01-induced anti-circumsporozoite antibodies and protection is consistent with data from other vaccine candidates and studies of naturally acquired immunity.^22^, ^23^, ^24^ However, such an association does not prove that anti-circumsporozoite antibodies cause protection.^25^ CD4-positive T cells also have a role in preventing infection,^26^, ^27^ but whether these cells act as direct effectors or indirectly through modulation of antibody responses is unclear. The lack of data on cell-mediated immunity is a potential limitation of this analysis. Analysis of the dynamics of anti-circumsporozoite antibodies after vaccination showed a biphasic pattern with rapid waning in the first 6 months followed by slower waning over the next 4 years. This pattern accords with waning of naturally acquired *P falciparum* antibody responses.^28^ The relatively short half-life of the long-lived component of the RTS,S-induced antibody response contrasts with vaccine-induced responses to other pathogens, which can have a much longer half-life.^29^, ^30^

The waning of efficacy after vaccination with RTS,S/AS01 makes characterisation of the duration of protection particularly important. Duration of efficacy has previously been estimated by point estimates for consecutive time windows.^2^, ^10^ Assessment of the waning of efficacy with a parametric form (the vaccine efficacy profile) enables robust estimation of duration without loss of statistical power because of the aggregation of data into time windows. This approach enables the incorporation of biologically relevant information on vaccine-induced immune responses. We predicted that the pattern of waning of efficacy against infection would mirror the dynamics of anti-circumsporozoite antibodies with rapid waning in the first 6 months followed by slower waning over the next 4 years. Efficacy against infection was not predicted to depend on transmission intensity because we assumed no acquisition of effective pre-erythrocytic immunity in young children.^31^ By contrast, lower levels of acquired blood-stage immunity in vaccinated compared with control participants means that those infections not prevented by vaccine-induced responses have a higher probability of progressing to episodes of clinical malaria. This effect causes the rate of waning of efficacy against clinical malaria to depend on transmission intensity.^10^ In low transmission areas, efficacy against clinical malaria wanes because of the reduction in anti-circumsporozoite antibody titres over time. In high transmission areas, efficacy against clinical malaria wanes more rapidly because of both the reduction in anti-circumsporozoite antibody titres and the lower levels of blood-stage immunity in vaccinated participants compared with control participants.

A combined analysis of the trial data incorporating anti-circumsporozoite antibodies and other covariates enables a detailed investigation of the results. For example, in the 6–12 week age category in Kintampo, efficacy against clinical malaria following primary vaccination was lower than in other sites (figure 2). Our analysis suggests that this finding might be partly explained by the low anti-circumsporozoite antibody titres in Kintampo, possibly because of high concentrations of maternally acquired antibodies before vaccination (table 1). This situation contrasts with the 5–17 month age category from Kintampo, in whom anti-circumsporozoite antibody titres were the highest of all trial sites with high levels of efficacy (figure 3).

In the event of a recommendation for vaccination of African children with RTS,S/AS01 with a booster dose, further analysis of the immunogenicity and efficacy of the booster will be crucial. The low anti-circumsporozoite antibody responses after the booster dose compared with primary vaccination suggests that the classic immunological picture of vaccine-induced responses being boosted to higher levels than after primary vaccination does not apply in the case of RTS,S/AS01. This finding might indicate shorter than usual half-lives for memory B cells or helper CD4-positive T cells. However, in the 5–17 month age category the long-lived component of the anti-circumsporozoite antibody response increased from 12% of the post-primary antibody response to 30% of the post-boost response, and the absolute titre of the long-lived response was higher after boost than after primary vaccination, suggesting there are longlasting benefits of the booster dose. The close concordance between data for clinical vaccine efficacy and anti-circumsporozoite antibody titres suggests that serological data might be used to assess future versions of RTS,S/AS01 and second generation anti-circumsporozoite vaccines, despite the limitations of extrapolating to other populations and vaccines. This approach will be much faster and more cost effective than running larger and larger efficacy trials to test new variations in dose, schedule, and adjuvant systems.

## Figures and Tables

**Table 1:** Malaria transmission intensity and anti-circumsporozoite antibody titres by site

	**Per-protocol cohort (C3C, R3C, R3R)**	**Incidence (cases per year)**	**Serology cohort (C3C, R3C, R3R)**	**CS**_base_**(EU/mL)**	**CS**_peak_**(EU/mL)**	**CS**_boost_**(EU/mL)**
**Age 6–12 weeks**						
Kilifi	102, 95, 90	0·04	104, 97, 87	0·39 (0·25–2·71)	247 (20–1326)	187 (11–1041)
Korogwe	183, 191, 191	0·09	74, 64, 64	0·34 (0·25–1·59)	232 (20–905)	163 (21–559)
Bagamoyo	245, 249, 252	0·15	70, 78, 74	0·34 (0·25–1·46)	163 (15–844)	149 (14–609)
Lambarene	62, 75, 72	0·17	64, 67, 72	0·28 (0·25–1·10)	283 (58–1374)	207 (15–1240)
Manhica	188, 187, 193	0·20	100, 107, 113	0·27 (0·25–0·70)	327 (46–1471)	254 (14–1141)
Lilongwe	257, 250, 247	0·42	104, 96, 99	0·39 (0·25–2·30)	218 (30–1213)	104 (7–668)
Agogo	221, 209, 209	0·84	70, 70, 63	0·46 (0·25–3·00)	151 (7–858)	128 (3–850)
Kombewa	196, 193, 195	1·62	68, 76, 75	0·38 (0·25–2·51)	202 (3–1250)	121 (2–876)
Kintampo	100, 101, 98	1·69	66, 69, 68	0·62 (0·25–3·20)	148 (24–829)	91 (0–591)
Siaya	229, 231, 221	3·12	91, 94, 94	0·46 (0·25–2·51)	208 (4–1442)	155 (0–1272)
Nanoro	224, 224, 217	3·14	65, 72, 64	0·94 (0·25–7·10)	115 (3–862)	156 (19–1101)
**Age 5–17 months**						
Kilifi	172, 172, 163	0·08	60, 72, 69	0·25 (0·25–0·25)	593 (41–2387)	231 (20–928)
Korogwe	293, 282, 286	0·10	66, 69, 66	0·25 (0·25–0·25)	540 (178–2098)	303 (130–965)
Bagamoyo	236, 242, 228	0·27	69, 68, 67	0·28 (0·25–1·59)	450 (52–1820)	297 (42–1417)
Lambarene	196, 196, 187	0·23	74, 78, 68	0·27 (0·25–0·86)	374 (55–1621)	193 (21–1121)
Manhica	*	*	81, 76, 76	0·26 (0·25–0·30)	621 (141–2315)	205 (36–834)
Lilongwe	185, 183, 176	0·23	70, 73, 77	0·26 (0·25–0·72)	360 (103–1630)	277 (65–747)
Agogo	191, 183, 188	1·01	69, 70, 68	0·28 (0·25–1·80)	667 (208–2703)	267 (89–909)
Kombewa	312, 301, 315	1·64	86, 65, 75	0·30 (0·25–1·41)	716 (204–2794)	306 (92–1386)
Kintampo	301, 310, 299	1·71	75, 71, 74	0·31 (0·25–1·91)	726 (112–2046)	260 (33–1339)
Siaya	252, 242, 240	3·15	93, 99, 89	0·37 (0·25–4·34)	677 (52–3154)	342 (67–1959)
Nanoro	198,195, 194	2·69	67, 70, 72	0·37 (0·25–4·24)	689 (184–3010)	499 (161–1922)

Cases are based on the primary case definition of clinical malaria: illness in a child brought to a study facility with a measured temperature of ≥37·5°C and *Plasmodium falciparum* asexual parasitaemia at a density of >5000 parasites per μL. Incidence is based on reported cases of malaria in the per-protocol population from 2·5 months to the end of the study. The serology cohort includes some children from the intention-to-treat population not included in the per-protocol population. CS antibody titres are presented as geometric mean titres with 95% ranges (2·5–97·5 percentile). CS=anti-circumsporozoite. R3C=three doses of RTS,S/AS01 and a booster with a comparator vaccine. R3R=three doses of RTS,S/AS01 and a booster with RTS,S/AS01. C3C=three doses of comparator vaccine and a booster with a comparator vaccine.

* No data in the per-protocol cohort.

**Table 2:** Determinants of immunogenicity of RTS,S

	**Primary schedule (n=2650)**		**Booster dose (n=1093)**	
	Estimate (95% CI)	p value	Estimate (95% CI)	p value
RTS,S (5–17 months): intercept	3·01 (2·91 to 3·10)	..	1·36 (1·08 to 1·65)	..
RTS,S (6–12 weeks)	−0·88 (−1·00 to −0·76)	<0·0001	−0·62 (−0·07 to −0·28)	<0·0001
Age (5–17 months)*	−0·015 (−0·022 to −0·009)	<0·0001	−0·006 (−0·015 to 0·003)	0·19
Age (6–12 weeks)*	0·022 (−0·038 to 0·081)	0·48	0·085 (−0·0002 to 0·174)	0·058
HIV positive	−0·53 (−0·64 to −0·42)	<0·0001	−0·22 (−0·51 to 0·07)	0·136
log_10_(CS_base_; 5–17 months)†	0·14 (0·05 to 0·24)	0·003	..	..
log_10_(CS_base_; 6–12 weeks)†	−0·58 (−0·70 to −0·46)	<0·0001	..	..
log_10_(CS_peak_; 5–17 months)†	..	..	0·42 (0·34 to 0·51)	<0·0001
log_10_(CS_peak_; 6–12 weeks)†	..	..	0·17 (0·06 to 0·29)	0·0025

Estimates from linear regression analyses of the effect of covariates on peak anti-circumsporozoite antibody titre after primary vaccination of RTS,S/AS01 (log_10_[CS_peak_/(EU/mL)]) or after a booster dose (log_10_[CS_boost_/(EU/mL)]). The intercept is taken to be vaccination of a child aged 5–17 months. Trial site was included in the regression models as a random effect. Transmission intensity, sex, preterm delivery, low weight-for-age *Z* score, and previous cases of clinical malaria were all tested as covariates but were not significant (appendix).

* Change associated with a 1 month change in age.

† Change associated with a ten-fold change in titre.

**Table 3:** Parameter estimates

	**Parameter**	**Prior**	**Posterior**	
			6–12 week category	5–17 month category
d_s_	Half-life of short-lived component of antibody response	46 days (43–49)	45 days (43–48)	45 days (42–48)
d_l_	Half-life of long-lived component of antibody response	572 days (269–1045)	634 days (574–709)	591 days (557–632)
ρ_peak_	Proportion of short-lived component following primary schedule	0·83 (0·63–0·95)	0·93 (0·92–0·94)	0·88 (0·87–0·89)
ρ_boost_	Proportion of short-lived component following booster dose	0·83 (0·63–0·95)	0·79 (0·77–0·81)	0·70 (0·68–0·72)
β	Scale parameter of dose–response curve	24·5 EU/mL (1·4–112·3)	99·2 EU/mL (67·6–132·6)	99·2 EU/mL (67·6–132·6)
α	Shape parameter of dose–response curve	0·92 (0·27–2·19)	0·74 (0·62–0·93)	0·74 (0·62–0·93)
V_max_	Maximum efficacy against infection	0·91 (0·74–0·99)	0·93 (0·83–0·99)	0·93 (0·83–0·99)

Parameter estimates for anti-circumsporozoite antibody dynamics and the dose–response relationship between antibody titres and efficacy against infection. Priors and posteriors are presented as median and 95% credible intervals. Informative priors are taken from phase 2 data.^6^
