# Supplementary material for: Immunogenicity of the RTS,S/AS01 malaria vaccine and implications for duration of vaccine efficacy: secondary analysis of data from a phase 3 randomised controlled trial
Source: Lancet Infect Dis. 2015 Dec;15(12):1450–8. doi: 10.1016/S1473-3099(15)00239-X (PMC4655306; doi:10.1016/S1473-3099(15)00239-X)
Supplement: Supplementary appendix [file mmc1.pdf]

THE LANCET Infectious Diseases

Supplementary webappendix

This webappendix formed part of the original submission and has been peer reviewed. We post it as supplied by the authors.

Supplement to: White MT, Verity R, Griffin JT, et al. Immunogenicity of the RTS,S/AS01 malaria vaccine and implications for duration of vaccine efficacy: secondary analysis of data from a phase 3 randomised controlled trial. *Lancet Infect Dis* 2015; published online Sept 2. http://dx.doi.org/10.1016/S1473-3099(15)00239-X.

Immunogenicity of the RTS,S/AS01 malaria vaccine over time and implications for duration of vaccine efficacy: analysis of data from a phase 3 randomised controlled trial

Michael T White*, Robert Verity, Jamie T Griffin, Kwaku Poku Asante, Seth Owusu-Agyei, Brian Greenwood, Chris Drakeley, Samwel Gesase, John Lusingu, Daniel Ansong, Samuel Adjei, Tsiri Agbenyega, Bernhards Ogutu, Lucas Otieno, Walter Otieno, Selidji T Agnandji, Bertrand Lell, Peter Kremsner, Irving Hoffman, Francis Martinson, Portia Kamthunzu, Halidou Tinto, Innocent Valea, Hermann Sorgho, Martina Okeno, Kephas Otieno, Mary J Hamel, Nahya Salim, Ali Mtoro, Salim Abdulla, Pedro Aide, Jahit Sacarlal, John J Aponte, Patricia Njuguna, Kevin Marsh, Philip Bejon, Eleanor M Riley, Azra C Ghani

* Corresponding author: m.white08@imperial.ac.uk

Supplementary Appendix: Statistical methods and extended results

This appendix provides full descriptions of all statistical and mathematical models used, as well as additional results and sensitivity analyses.

A note on statistical methods.

The analysis of determinants of immunogenicity (Section 2) was undertaken using frequentist linear regression models. Results are presented with 95% confidence intervals (CI). The analyses of antibody dynamics (Section 3) and the association between antibodies and protection (Section 4) were undertaken in a Bayesian framework, and results are therefore presented as posterior medians with 95% credible intervals (CrI). To account for variation between individuals, measurements of antibody titres are presented as geometric mean titres (GMT) with 95% ranges. Note that statistical inference for the antibody dynamics model (Section 3) and the model for the association between antibodies and protection (Section 4) were fitted to the data in a two stage process, with estimates of antibody dynamics over time incorporated into the model for the incidence of clinical malaria.

1. Overview of data from the control cohort

The data on incidence of clinical malaria in the control cohort provides valuable information on the intensity and seasonality of malaria transmission in each trial site. Figure S1 shows the recorded cases of malaria broken down by time and trial site for the according-to-protocol (ATP) population over the period from month 2·5 to study end. There was a large degree of variation in transmission intensity and seasonality between sites. The primary case definition of an episode of clinical malaria is used: illness in a child brought to a study facility with a measured temperature of ≥37·5°C or reported fever within the last 24 hours and *P. falciparum* asexual parasitaemia at a density of >5000 parasites per cubic millimetre. This parasite threshold is within a sufficient range to ensure optimal statistical power in studies of the efficacy of pre-erythrocytic vaccines[1].

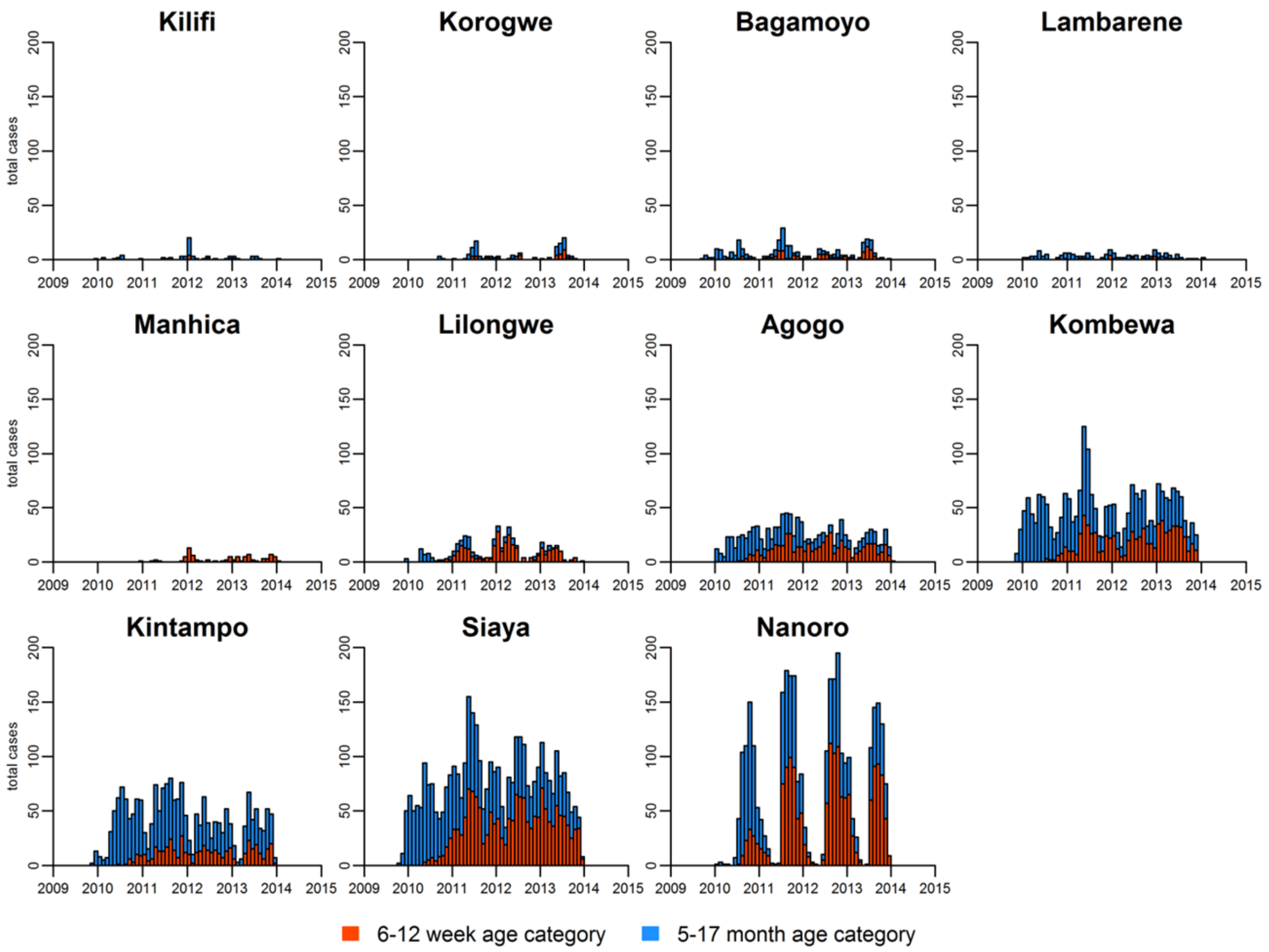

Figure S1: Total numbers of cases of clinical malaria (primary case definition) in the control arm for each of the 11 trial sites.

In addition to heterogeneity between sites, there was substantial heterogeneity within sites in the incidence of clinical of malaria, with some children experiencing a large number of episodes and many children not experiencing any episodes over the duration of the trial. The distribution of episodes of clinical malaria within each trial site is shown for the 6-12 week age category (Figure S2) and for the 5-17 month age category (Figure S3).

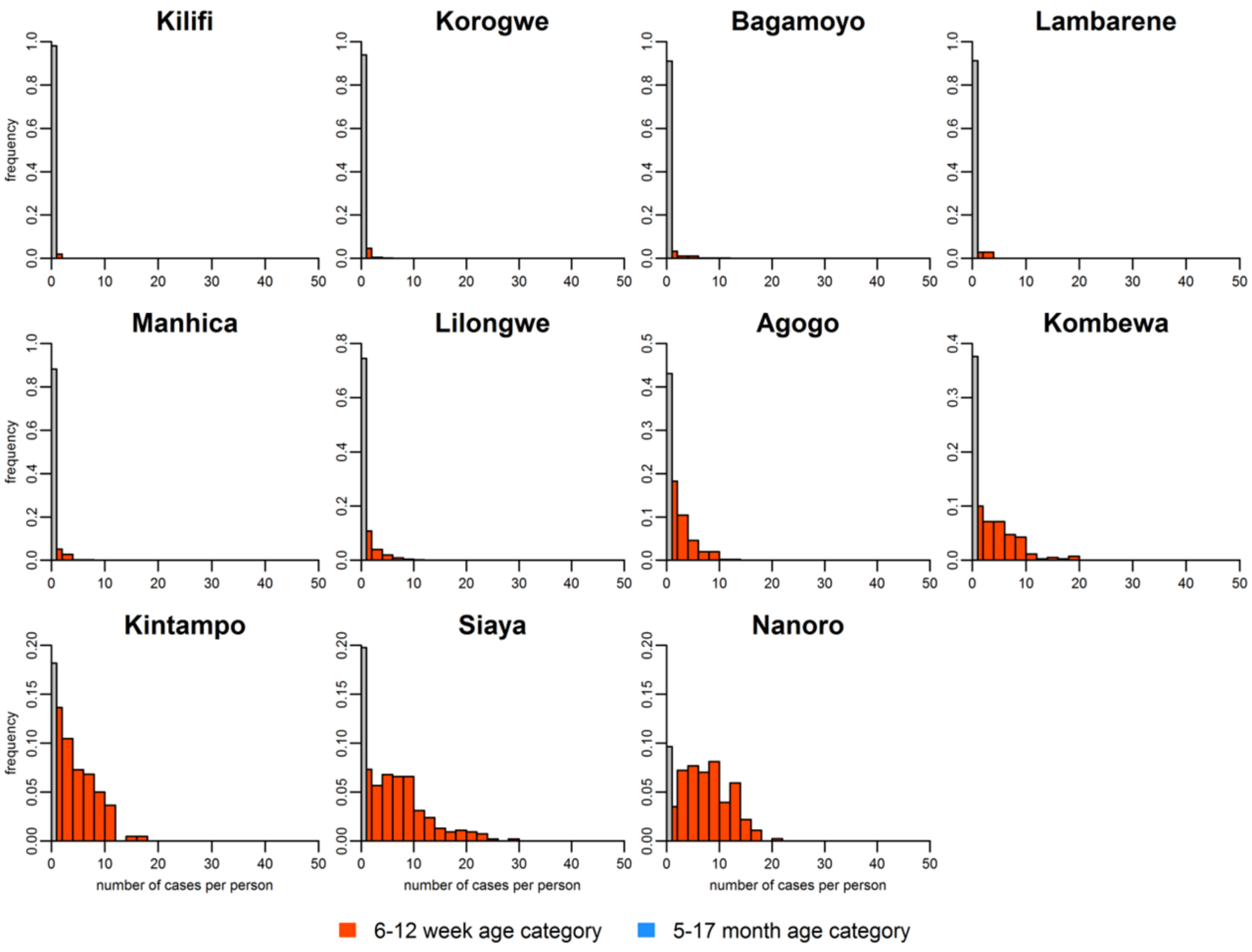

Figure S2: Distribution of number of cases of clinical malaria (primary definition) per individual in the control arm of the 6-12 week age category. The grey bar denotes individuals with no recorded cases. The average time at risk is approximately 3 years although there is variation between individuals in the length of follow-up.

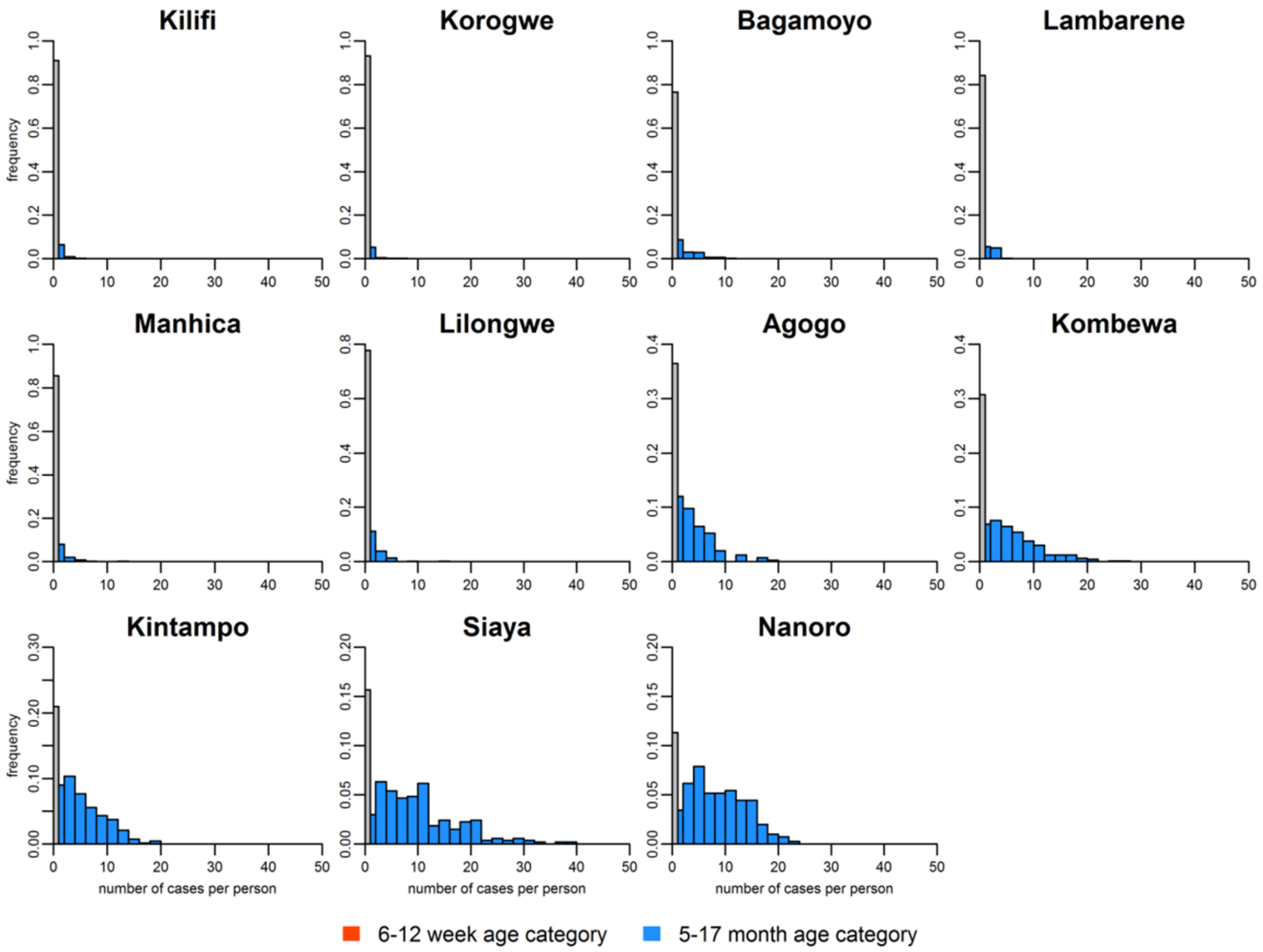

Figure S3: Distribution of number of cases of clinical malaria (primary definition) per individual in the control arm of the 5-17 month age category. The grey bar denotes individuals with no recorded cases. The average time at risk is approximately 3·5 years although there is variation between individuals in the length of follow-up.

As illustrated above, the incidence of clinical malaria in the control cohort depends on site-level transmission intensity and seasonality. The incidence of clinical malaria also depends on age. This is due to the acquisition of clinical immunity over time as children are exposed to malaria[2, 3]. In addition, as children grow older they are exposed to a greater number of mosquito bites due to their increased body size[4, 5]. Figure S4 shows the age-incidence curves for each trial site based on reported cases of malaria (primary case definition) in the control cohort.

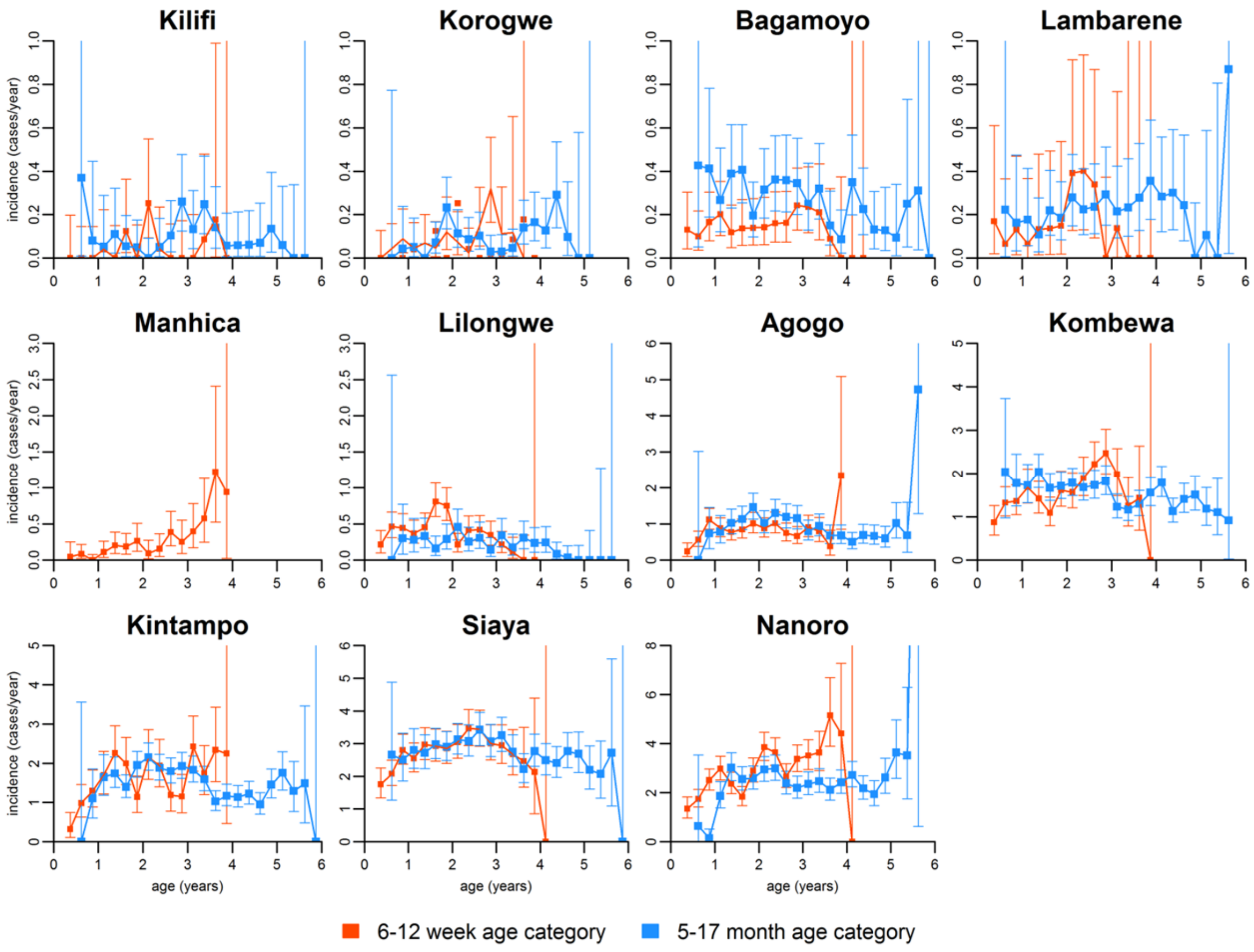

Figure S4: Age-incidence curves of clinical malaria (primary definition) in the control cohort for the according-to-protocol (ATP) population. Clinical incidence was calculated as the number of cases in 3 month age bins divided by the time at risk in each bin. Edge effects due to small sample sizes are apparent in some cases. In sites with higher transmission intensity an increase in cases is apparent over the first two years. There is evidence of decreasing transmission intensity in two sites (Bagamoyo and Lilongwe).

2. Determinants of immunogenicity

2.1. Overview of immunogenicity data

The immunogenicity of RTS,S/AS01 was evaluated via its capacity to induce anti-CS antibody titres following a primary vaccination schedule (CS_{peak}) or following a booster dose (CS_{boost}). The magnitude of anti-hepatitis B (HBs) antibody titres induced following vaccination with RTS,S/AS01 (HBs_{peak}) also provides valuable information on immunogenicity. Phase 2 trials of the RTS,S/AS01 and RTS,S/AS02 vaccines have demonstrated that peak anti-CS antibody titres following a primary schedule of RTS,S depend on a number of covariates such as age and baseline antibody titres[6]. Notable findings from phase 2 trials included:

- Children (aged > 3 months and < 5 years) had higher antibody responses than infants (aged < 3 months)[7, 8].
- RTS,S/AS01 induced higher antibody responses than RTS,S/AS02[9, 10].
- Receiving 3 doses of RTS,S induced higher antibody responses than 2 doses.
- In infants, high baseline anti-CS antibody titres were associated with lower peak anti-CS antibody titres post-vaccination. This may be attributable to maternally acquired antibodies inhibiting the vaccine-induced antibody response[11].
- In children, baseline anti-HBs antibody titres (most likely due to prior Hepatitis B vaccination) were associated with higher peak anti-CS antibody titres[12].

Data from the phase 3 trials of the RTS,S/AS01 vaccine were analysed to investigate the determinants of immunogenicity following primary vaccination with or without a booster dose. Anti-CS antibodies were measured by ELISA in the first 200 participants in each age category at each study site at enrolment and 1 month after the third dose of vaccine. This assay was based on the binding of serum antibodies to R32LR, a recombinant protein composed of the repeat region of *P. falciparum* CS. The threshold for a positive titer was 0.5 EU/ml[13]. Figure S5 shows the dependence on age at first dose of anti-CS antibody titres at baseline (CS_{base}), following primary vaccination (CS_{peak}) and following a booster dose (CS_{boost}). Figure S6 and Figure S7 show the associations between anti-CS and anti-HBs antibody titres at baseline, following primary vaccination and following a booster dose in the 6-12 week and 5-17 month age categories respectively.

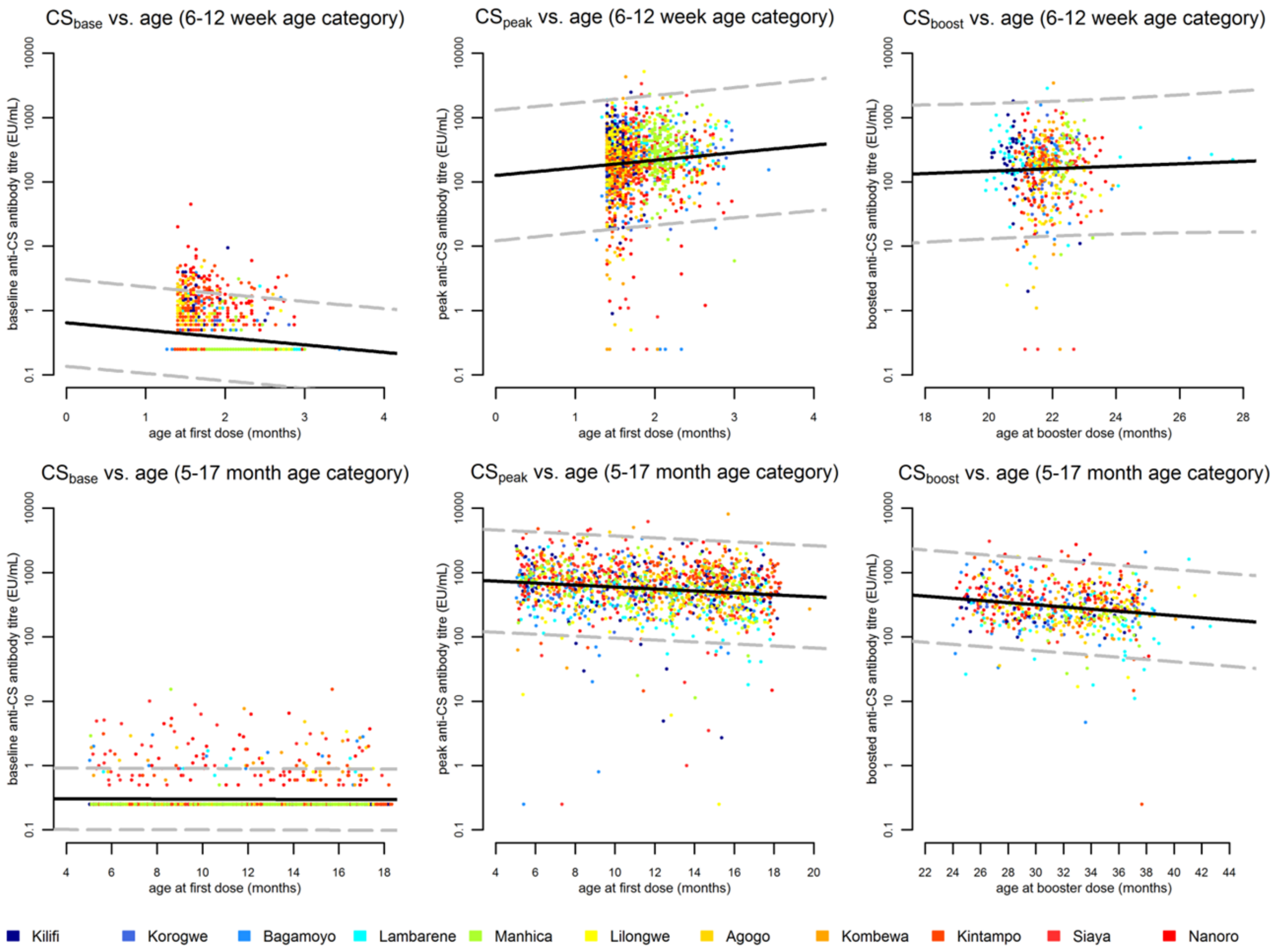

Figure S5: Age-dependency of antibody titres.

Dependence on age at first dose of baseline anti-CS antibody titres (CS_{base}), anti-CS antibody titres following a primary schedule of RTS,S/AS01 (CS_{peak}) and anti-CS antibody titres following a booster dose (CS_{boost}). The black lines show the association between antibody titres and age predicted by linear regression models. The dashed grey lines depict the variation in the data.

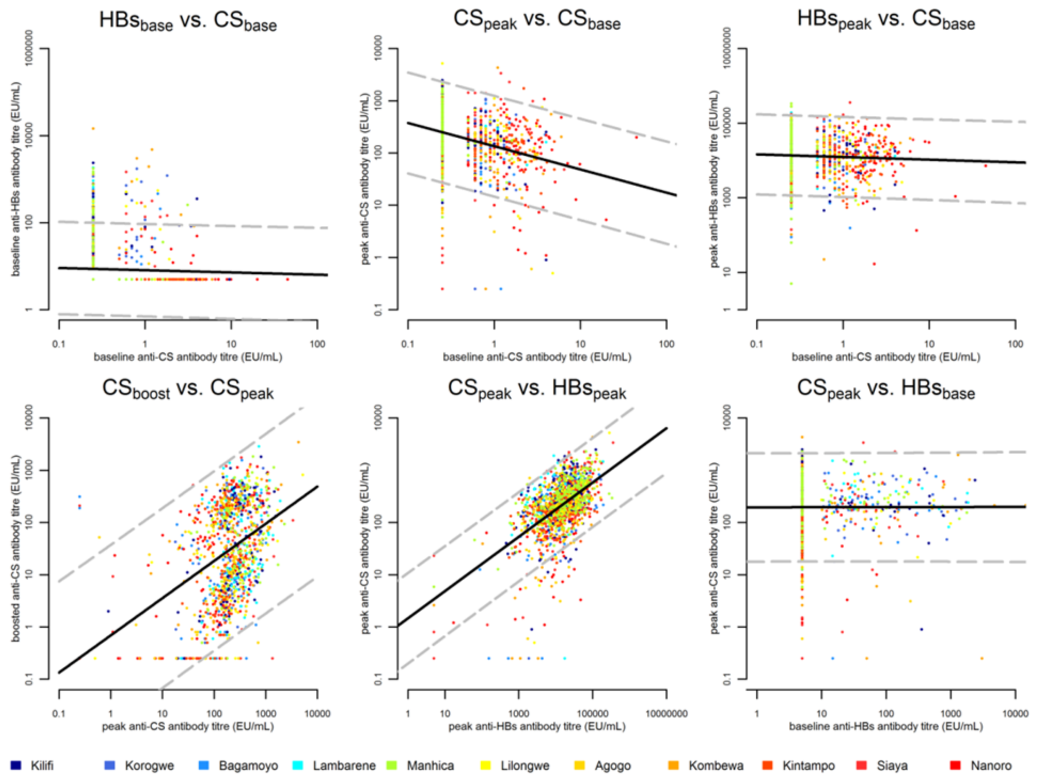

Figure S6: Associations between antibody titres in the 6-12 week age category.
Association between anti-circumsporozoite (CS) and anti-hepatitis B (HBs) titres at baseline, following primary schedule of RTS,S/AS01 with or without a booster dose. The black lines show the relationships predicted by linear regression models. The dashed grey lines depict the variation in the data.

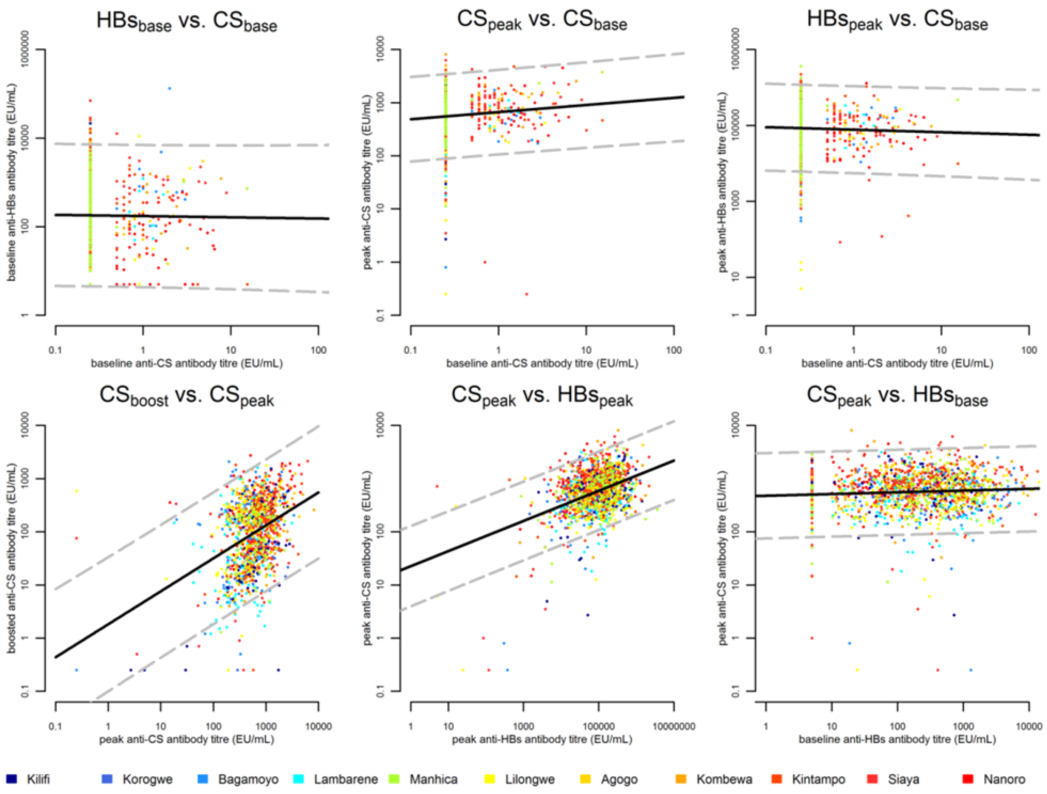

Figure S7: Associations between antibody titres in the 5-17 month age category.
Association between anti-circumsporozoite (CS) and anti-hepatitis B (HBs) titres at baseline, following primary schedule of RTS,S/AS01 with or without a booster dose. The black lines show the relationships predicted by linear regression models. The dashed grey lines depict the variation in the data.

2.2. Statistical analysis of immunogenicity data

The immunogenicity of RTS,S/AS01 (as measured by anti-CS antibody titres following primary vaccination or a booster dose) was analysed to investigate the dependence on a number of covariates. Covariates considered included:

- age category (6-12 weeks or 5-17 months)
- age at first dose (in months)
- HIV status (confirmed positive)
- gender
- site-level transmission intensity (based on incidence of clinical malaria in the control cohort)
- pre-term delivery
- very low weight-for-age z-score
- previous cases of malaria (analysis of booster data only)
- CS_{base}: anti-CS antibody titre at screening
- HBs_{base}: anti-HBs antibody titre at screening
- CS_{peak}: peak anti-CS antibody titre (analysis of booster data only)

The data were analysed using linear regression models with trial site included as independent random effects. Table S1 shows the results of the linear regression models with all covariates included. Table 2 of the main manuscript shows the results of a linear regression model with some of the non-significant covariates removed. A number of statistically significant associations are evident for peak anti-CS antibody titres following a primary schedule of RTS,S/AS01:

- Children in the 5-17 month age category had higher antibody responses than infants in the 6-12 week age category.
- Within the 5-17 month age category age was negatively associated with anti-CS antibody response.
- HIV infection was associated with significantly lower anti-CS antibody responses.
- Pre-term delivery or very low weight-for-age z-scores were not associated with impaired anti-CS antibody responses.
- In the 6-12 week age category, pre-vaccination anti-CS antibody titres were associated with lower peak anti-CS antibody titres. This finding is consistent with the hypothesis that maternal antibodies reduce the immunogenicity of RTS,S[11].
- No statistically significant associations were detected between pre-vaccination anti-HBs antibodies and peak anti-CS antibody titres. This finding is in contrast to results from phase 2 trials which found baseline anti-HBs titres to be positively associated with RTS,S immunogenicity[12]. However, these findings are consistent with the hypothesis that it is prior hepatitis B vaccination status and not the magnitude of the anti-HBs antibody

titre that affects RTS,S immunogenicity. Note that all children and infants in the phase 3 trial of RTS,S/AS01 received prior anti-hepatitis B vaccination.

The data from individuals in the R3R cohort were analysed to investigate the determinants of immunogenicity following a booster dose of RTS,S/AS01 (Table 2 and Table S1). A number of statistically significant associations are evident:

- The most significant predictor of boosted anti-CS antibody titre is peak anti-CS antibody titre.
- There were no statistically significant associations between age at first dose and boosted anti-CS antibody titres at the 5% significance level. At the 10% level, increased age was associated with lower boosted anti-CS antibody titre in the 5 to 17 month cohort. Increased age at first dose was associated with higher boosted anti-CS antibody titre in the 6 to 12 week cohort.
- There was no statistically significant association between boosted anti-CS antibody titres and recent malaria exposure (as measured by the number of cases of clinical malaria between dose 3 and the booster dose). A negative association may have indicated immunosuppression by malaria infection.

Table S1: Determinants of immunogenicity of RTS,S/AS01 including non-significant covariates.
Estimates from linear regression analyses of the impact of covariates on peak anti-CS antibody titre following primary vaccination of RTS,S/AS01 ($\log_{10}(CS_{peak} / (EU/mL))$) or following a booster dose ($\log_{10}(CS_{boost} / (EU/mL))$). The baseline is taken to be vaccination of a child in the 5-17 month age category. Trial site was included in the regression models as a random effect. Transmission intensity was accounted for using the incidence of cases of clinical malaria in the control cohort of the 6-12 week age category (Table 1). The number of cases of clinical malaria between dose 3 and the booster dose was counted as cases before booster. ¶ indicates the change associated with a one month change in age. † indicates the change associated with a 10-fold change in titre.

	**primary schedule (N=2650)**		**booster dose (N = 1093)**	
	estimate (95% CI)	***P value***	***estimate (95% CI)***	***P value***
RTS,S (5-17 months): intercept	2·95 (2·82, 3·09)		1·46 (1·15, 1·78)	
RTS,S (6-12 weeks)	-0·83 (-0·99, -0·68)	< 0·00001	-0·73 (-1·10, -0·36)	< 0·00001
age*(5-17 months) ¶	-0·015 (-0·021, -0·009)	< 0·00001	-0·008 (-0·018, 0·001)	0·0911
age*(6-12 weeks) ¶	0·022 (-0·039, 0·082)	0·48	0·085 (-0·003, 0·174)	0·0593
HIV positive	-0·53 (-0·64, -0·42)	< 0·00001	-0·23 (-0·52, 0·06)	0·118
gender	-0·03 (-0·06, 0·01)	0·089	-0·03 (-0·08, 0·02)	0·24
transmission intensity	0·026 (-0·013, 0·064)	0·22	0·01 (-0·03, 0·05)	0·29
pre-term delivery	0·007 (-0·097, 0·112)	0·89	0·09 (-0·08, 0·27)	0·63
low weight-for-age score	-0·07 (-0·20, 0·11)	0·27	0·10 (-0·09, 0·29)	0·31
cases before booster			0·010 (-0·007, 0·026)	0·27
$\log_{10}(CS_{base})$*(5-17 months) †	0·13 (0·04, 0·23)	0·003		
$\log_{10}(CS_{base})$*(6-12 weeks) †	-0·58 (-0·70, -0·46)	< 0·00001		
$\log_{10}(CS_{peak})$*(5-17 months) †			0·42 (0·33, 0·51)	< 0·00001
$\log_{10}(CS_{peak})$*(6-12 weeks) †			0·18 (0·07, 0·29)	0·0018
$\log_{10}(HBs_{base})$*(5-17 months) †	0·015 (-0·015, 0·044)	0·38	-0·03 (-0·07, 0·01)	0·16
$\log_{10}(HBs_{base})$*(6-12 weeks) †	-0·025 (-0·080, 0·030)	0·37	0·02 (-0·06, 0·10)	0·66

The effect of RTS,S/AS01 on anti-hepatitis B antibody titres following vaccination was also analysed using linear regression models (Table S2). RTS,S/AS01 was more immunogenic in the 5-17 month age category (GMT = 83472 (95% range: 5203, 681620) EU/mL) compared to the 6-12 week age category (GMT = 13474 (95% range: 1014, 98408) EU/mL).

Table S2: Determinants of RTS,S-induced anti-hepatitis B (HBs) antibody titres.
Estimates from linear regression analyses of the impact of covariates on peak anti-HBs antibody titre following primary vaccination of RTS,S/AS01 ($\log_{10}(HBs_{peak} / (EU/mL))$). The baseline is taken to be vaccination of a child in the 5-17 month age category. Trial site was included in the regression models as a random effect. Transmission intensity was accounted for using the incidence of cases of clinical malaria in the control cohort of the 6-12 week age category (Table 1). ¶ indicates the change associated with a one month change in age. † indicates the change associated with a 10-fold change in titre.

	**primary schedule (N=2640)**	
	estimate (95% CI)	***P value***
RTS,S (5-17 months): intercept	3·74 (3·58, 3·90)	
RTS,S (6-12 weeks)	0·38 (0·20, 0·56)	< 0·00001
age*(5-17 months) ¶	0·030 (0·023, 0·038)	< 0·00001
age*(6-12 weeks) ¶	-0·007 (-0·076, 0·064)	0·86
HIV positive	-0·64 (-0·77, -0·52)	< 0·00001
gender	-0·039 (-0·078, 0·001)	0·054
transmission intensity	0·009 (-0·033, 0·051)	0·69
pre-term delivery	-0·073 (-0·19, 0·048)	0·24
low weight-for-age score	0·06 (-0·09, 0·20)	0·43
$\log_{10}(CS_{base})$*(5-17 months) †	-0·05 (-0·15, 0·06)	0·41
$\log_{10}(CS_{base})$*(6-12 weeks) †	-0·03 (-0·17, 0·10)	0·62
$\log_{10}(HBs_{base})$*(5-17 months) †	0·38 (0·35, 0·41)	< 0·00001
$\log_{10}(HBs_{base})$*(6-12 weeks) †	-0·41 (-0·47, -0·34)	< 0·00001

The variation in peak and boosted anti-CS and anti-HBs antibody titres between trial sites is only partially explained by the covariates tested above, and must instead be accounted for through the site-specific random effects. It is possible that this variation may be explained by a multitude of other unmeasured factors such as host-specific genetic variation, nutrition, or immunosuppression due to helminth infection[14].

3. Anti-CS antibody dynamics

3.1. Antibody dynamics model

The patterns of waning of anti-CS antibody titres following RTS,S/AS01 vaccination without (R3C) and with (R3R) a booster dose at 18 months are shown in Figure S8. Also shown is the anti-CS antibody titres over time for members of the control cohort (C3C). Anti-CS GMTs in the control cohort are orders of magnitude lower than anti-CS GMTs in both of the vaccine cohorts throughout follow-up.

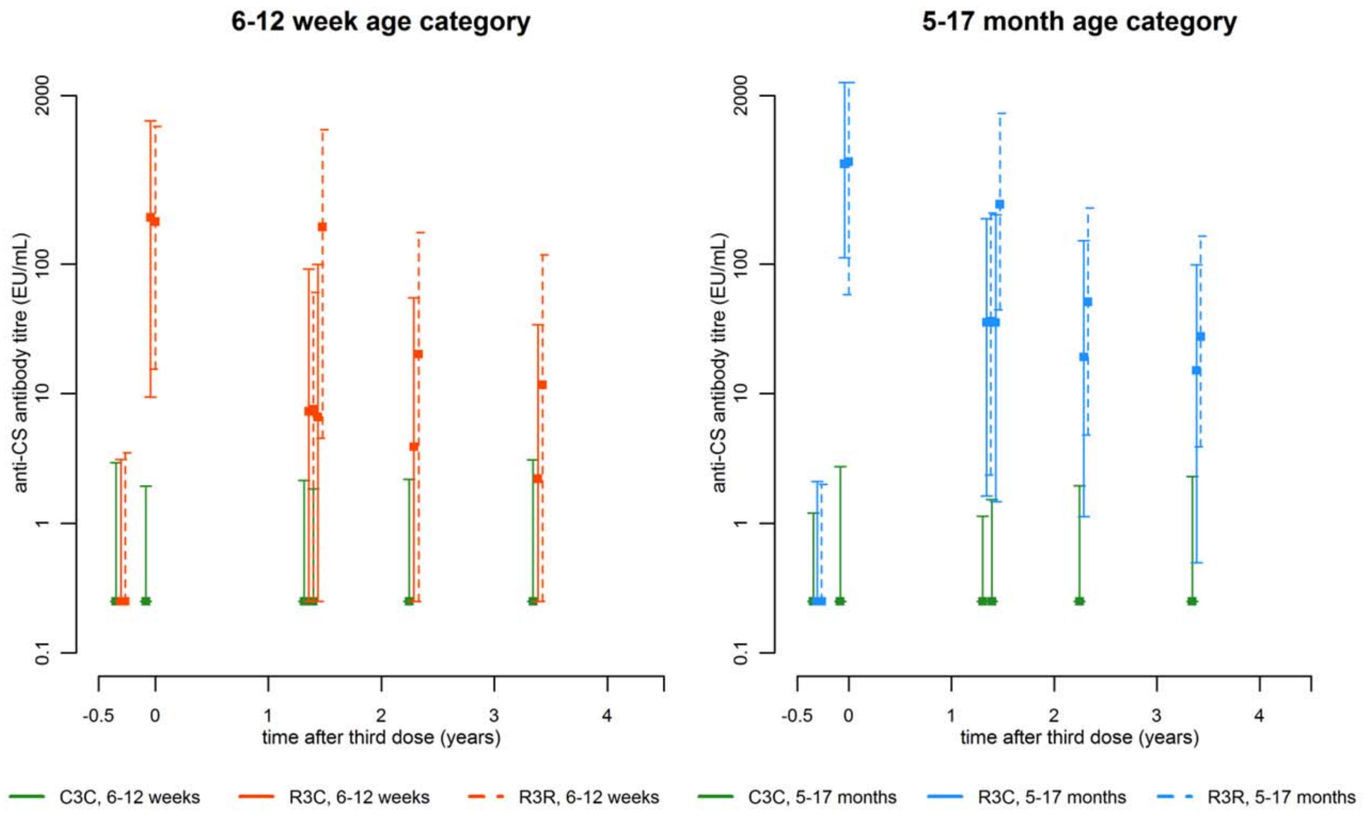

Figure S8: Overview of measured anti-CS antibody titres in both age categories. Data are shown as geometric mean titres (GMT) with 95% ranges. Data from the control cohort (C3C) are shown in green. Data from the booster cohort (R3R) are shown with dashed lines.

Following vaccination with a primary schedule of RTS,S/AS01, anti-CS antibody titres are assumed to increase to CS_{peak} and then wane over time *t* according to a bi-phasic exponential model as follows:

$$CS(t) = CS_{\text{peak}} \left(\rho_{\text{peak}} e^{-r_s t} + (1-\rho_{\text{peak}}) e^{-r_l t} \right) \quad \text{(S1)}$$

where $r_s = \log(2)/d_s$ and $r_l = \log(2)/r_l$ are the decay rates of the short-lived and long-lived components of the antibody response, and ρ_{peak} is the proportion of the antibody response that is short-lived. d_s and d_l are the half-lives of the short-lived and long-lived components of the antibody response. Following a booster dose of RTS,S/AS01 at time t_{boost} anti-CS antibody titres increase to CS_{boost}. It is assumed that the rate of decay of the short-lived and long-lived components of the antibody response remains the same, but that the proportion of the response that is short-lived ρ_{boost} may change. For $t > t_{boost}$ the antibody dynamics can be described by the following equation:

$$CS(t) = CS_{\text{boost}}\left(\rho_{\text{boost}} e^{-r_s(t-t_{\text{boost}})} + (1-\rho_{\text{boost}}) e^{-r_l(t-t_{\text{boost}})}\right) \quad \text{(S2)}$$

Figure S9 shows a schematic representation of how the booster dose can be incorporated into a model of anti-CS antibody dynamics. Two model formulations are considered.

- Model 1: the dynamics following the booster dose are governed by the same equation as the dynamics following the primary schedule.
- Model 2: following the booster dose, the long-lived component of the antibody response is comprised of antibodies generated in response to both the primary schedule and the booster dose.

The two model formulations are mathematically equivalent under the conditions that $CS_{\text{boost}}\rho_{\text{boost}} \geq CS_{\text{peak}}\rho_{\text{peak}} e^{-r_s t_{\text{boost}}}$ and $CS_{\text{boost}}\left(1-\rho_{\text{boost}}\right) \geq CS_{\text{peak}}\left(1-\rho_{\text{peak}}\right) e^{-r_l t_{\text{boost}}}$. Therefore we choose the mathematically simpler Model 1 for fitting to the data.

For a participant with serological data, CS_{peak} and CS_{boost} can be measured directly from the data. It is also possible to obtain estimates of CS_{peak} and CS_{boost} using the regression models from section 2 and data on covariates such as age and pre-vaccination antibody titres. However, we prefer to directly use the measured antibody titres to avoid the uncertainty in the regression models from affecting the antibody dynamics models.

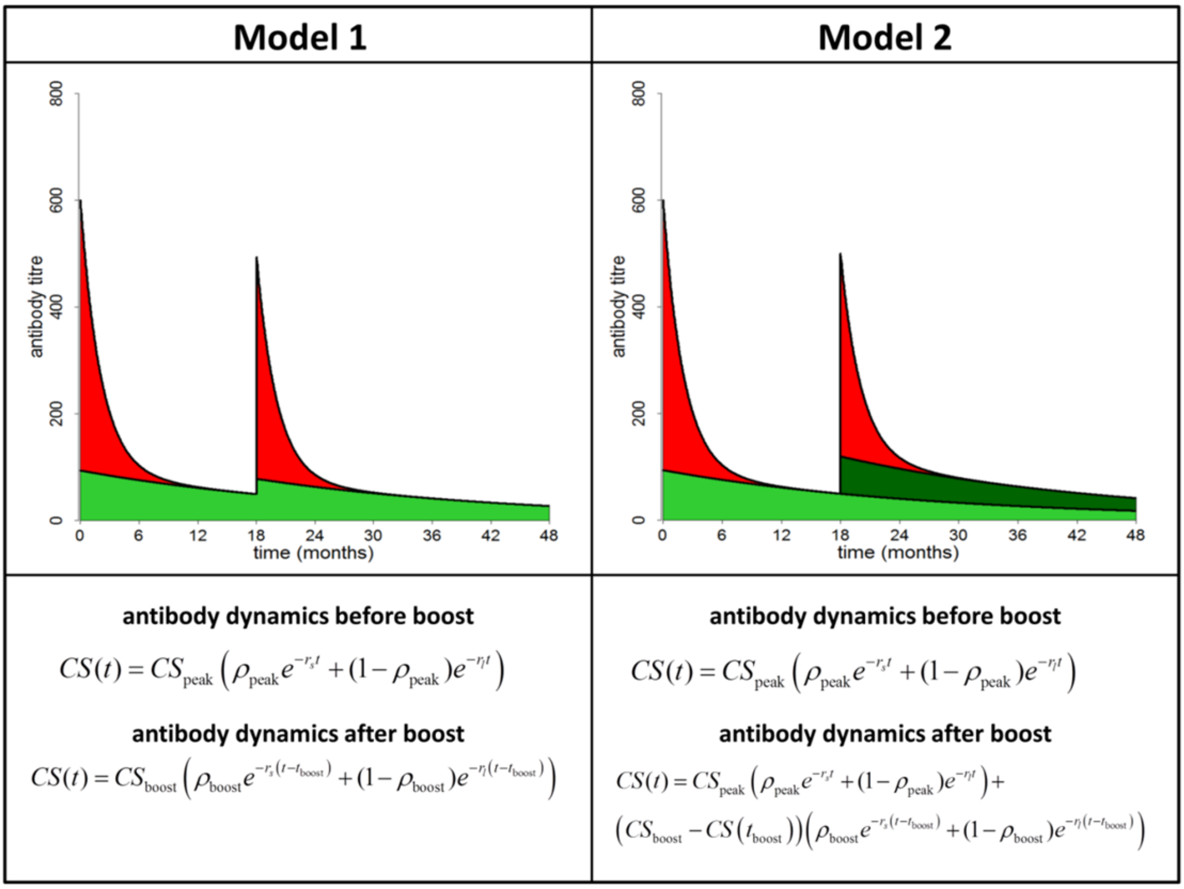

Figure S9: Schematic representation of anti-CS antibody dynamics following a primary schedule and a booster dose of RTS,S/AS01. The short-lived component of the antibody response is indicated in red. The long-lived component of the antibody response is indicated in green. For Model 2 the long-lived responses generated by the primary and booster doses are indicated separately.

3.2. Fitting the model to data

The model was fitted to longitudinal antibody titre measurements from vaccinated participants in each study site. Mixed effects methods were used to capture the natural variation in antibody dynamics between individual participants, whilst estimating the average value and variance of the immune parameters across the entire cohort of children[15]. The models were fitted in a Bayesian framework using Markov Chain Monte Carlo (MCMC) methods. Mixed effects methods allow local parameters to be estimated for each child individually, with these local (or mixed effects) parameters being drawn from global distributions[16]. For example, for each participant *n* the half-life of the short-lived component of the antibody response may be estimated as d_s^n (an individual specific parameter). These *N* estimates of the local parameters d_s^n will be drawn from a probability distribution. A Log-Normal distribution is suitable as it has positive support on $[0,\infty)$. Thus we have $\log(d_s^n) \sim N\left(\mu_s, \sigma_s^2\right)$. The mean d_s and the variance Σ_s^2 of the estimates of d_s^n are given by $d_s = e^{\mu_s + \frac{\sigma_s^2}{2}}$ and $\Sigma_s^2 = \left(e^{\sigma_s^2} - 1\right) e^{2\mu_s + \sigma_s^2}$. The relationship between the global parameters describing the population level distribution and the local parameters for each individual in the population are depicted in the schematic diagram in Figure S9.

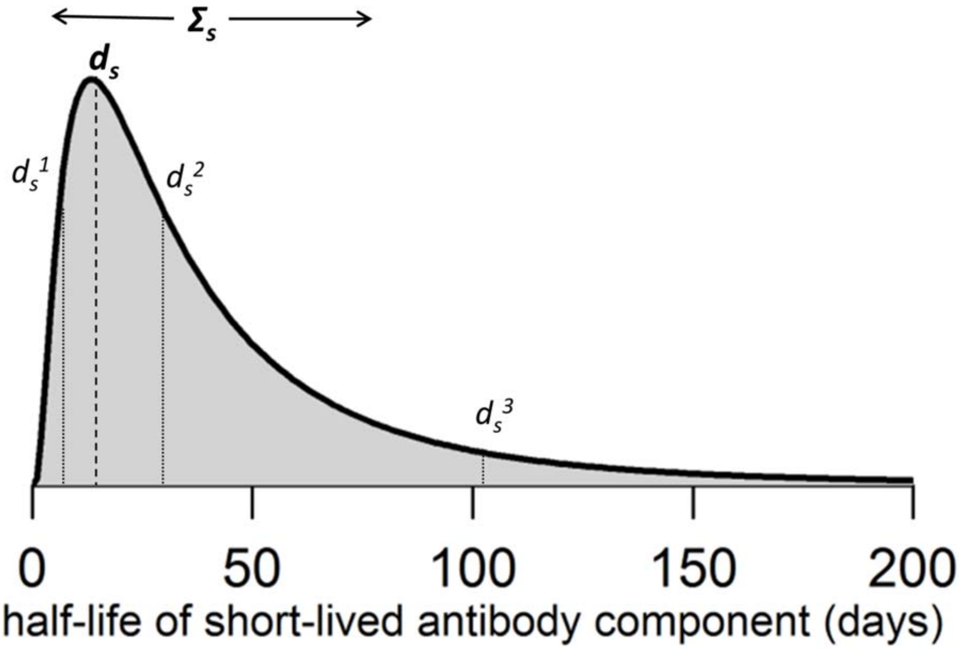

Figure S10: Schematic representation of the relationship between the global and local parameters for the half-life of the short-lived component of the vaccine-induced antibody response. It is assumed that half-lives are log-Normally distributed throughout the population. The global parameters define the mean d_s and standard deviation Σ_s of this distribution. The local parameters d_s^n for each of the *n* participants follow the log-Normal distribution defined by the global parameters. Three representative local parameters are shown for illustration.

Model likelihood

For participant *n* we have data on observed antibody titres $\mathrm{A}^n = \{a_1, ..., a_J\}$ at times $T^n = \{t_1, ..., t_J\}$. We denote $D^n = \left(A^n, T^n\right)$ to be the vector of data for participant *n*. For participant *n*, the four parameters d_s^n, d_l^n, ρ_{peak}^n and ρ_{boost}^n are estimated. These parameters are denoted $\theta^n = \left(d_s^n, d_l^n, \rho_{\text{peak}}^n, \rho_{\text{boost}}^n\right)$. The model predicted antibody titres will be $\{CS(t_1), CS(t_2), ..., CS(t_J)\}$. We assume log-Normally distributed measurement error such that the difference between log(a_j) and $\log\left(CS(t_j)\right)$ is Normally distributed with variance σ_{obs}^2. For model predicted antibody titres $CS(t_j)$ the data likelihood for participant *n* is given by

$$L_{\text{mod}}^n\left(\theta^n \middle| D^n\right) = \prod_{j \in J} \frac{e^{-\frac{\left(\log(a_j) - \log(CS(t_j))\right)^2}{2\sigma_{\text{obs}}^2}}}{a_j \sigma_{\text{obs}} \sqrt{2\pi}} \tag{S3}$$

Mixed effects likelihood

There are four mixed effects parameters to be estimated: d_s, d_l, ρ_{peak} and ρ_{boost}. The mixed effects likelihood is thus

$$L_{\text{mix}}^n\left(\theta^n \middle| D^n\right) = \left(\frac{e^{-\frac{(\log(d_s^n) - \mu_s)^2}{2\sigma_s^2}}}{\sqrt{2\pi} d_s^n \sigma_s}\right)\left(\frac{e^{-\frac{(\log(d_l^n) - \mu_l)^2}{2\sigma_l^2}}}{\sqrt{2\pi} d_l^n \sigma_l}\right)\left(\frac{e^{-\frac{(\log(\frac{\rho_{\text{peak}}^n}{1-\rho_{\text{peak}}^n}) - \mu_{\rho,\text{peak}})^2}{2\sigma_{\rho,\text{peak}}^2}}}{\sqrt{2\pi}\rho_{\text{peak}}^n(1-\rho_{\text{peak}}^n)\sigma_{\rho,\text{peak}}}\right)\left(\frac{e^{-\frac{(\log(\frac{\rho_{\text{boost}}^n}{1-\rho_{\text{boost}}^n}) - \mu_{\rho,\text{boost}})^2}{2\sigma_{\rho,\text{boost}}^2}}}{\sqrt{2\pi}\rho_{\text{boost}}^n(1-\rho_{\text{boost}}^n)\sigma_{\rho,\text{boost}}}\right) L_{\text{mod}}^n\left(\theta^n \middle| D^n\right) \tag{S4}$$

As the proportion of the antibody response that is short-lived must be bounded by 0 and 1, the local parameters ρ_{peak}^n and ρ_{boost}^n are assumed to be drawn from a Logit-Normal distribution. Thus for example $\log(\frac{\rho^n}{1-\rho^n}) \sim N\left(\mu_\rho, \sigma_\rho^2\right)$. In addition, this formulation of the likelihood assumes that parameters are independent and not correlated.

Total model likelihood

Denote $D = \{D^1, ..., D^N\}$ to be the vector of data for all *N* participants. We denote $\theta = \left(d_s, d_l, \rho_{\text{peak}}, \rho_{\text{boost}}, \sigma_s, \sigma_l, \sigma_{\rho,\text{peak}}, \sigma_{\rho,\text{boost}}, \sigma_{\text{obs}}, \theta^1, ..., \theta^N\right)$ to be the combined vector of global parameters and local parameters to be estimated. The total likelihood is obtained by multiplying the likelihood for each child

$$L_{\text{total}}\left(\theta \middle| D\right) = \prod_{n \in N} L_{\text{mix}}^n\left(\theta^n \middle| D^n\right) \tag{S5}$$

Markov Chain Monte Carlo parameter update

The model was fitted to the data using Markov Chain Monte Carlo (MCMC) methods. Parameters were updated at each MCMC iteration using a random walk Metropolis-Hastings algorithm with two update stages illustrated below. A ′ indicates an attempted update.

1. **Local parameter update.** For each participant *n*:
   - Update local parameters: $\theta^{n\prime} = \left(d_s^{n\prime}, d_l^{n\prime}, \rho_{\text{peak}}^{n\prime}, \rho_{\text{boost}}^{n\prime} \right)$
   - Calculate updated mixed effects likelihood $L_{\text{mix}}^{n}\left(\theta_n' \middle| D_n\right)$
   - Accept the parameter update with probability $\min\left(1, \frac{L_{\text{mix}}^{n}\left(\theta_n' \middle| D_n\right)}{L_{\text{mix}}^{n}\left(\theta_n \middle| D_n\right)}\right)$

2. **Global parameter update.**
   - Update global parameters: $\theta' = \left(d_s', d_l', \rho_{\text{peak}}', \rho_{\text{boost}}', \sigma_s', \sigma_l', \sigma_{\rho,\text{peak}}', \sigma_{\rho,\text{boost}}', \sigma_{\text{obs}}', \theta^1, \ldots, \theta^N \right)$
   - Calculate updated total likelihood $L_{\text{total}}\left(\theta' \middle| D\right)$ and the updated prior probability density $P\left(\theta'\right)$
   - Accept the parameter update with probability $\min\left(1, \frac{L_{\text{total}}\left(\theta' \middle| D\right) P\left(\theta'\right)}{L_{\text{total}}\left(\theta \middle| D\right) P\left(\theta\right)}\right)$

All updates were attempted with Normal proposal distributions. The MCMC algorithm was implemented in C++. The variances of the proposal distributions were first estimated by performing 1 million MCMC iterations to estimate the variance of the posterior distributions. 20 million MCMC iterations were computed with calibration of acceptance rates using a Robbins-Munro algorithm[17]. All Markov chains were visually examined for appropriate mixing and convergence. Such large numbers of iterations were needed because of the large number of parameters to be estimated. Convergence was formally tested for using the Gelman-Rubin statistic[18], which was found to be less than 1.05 in all cases suggesting adequate convergence. The effective number of iterations was calculated using the effectiveSize routine in the R library coda[19] and the effective size was checked to be > 1,000 for the global parameters. The MCMC fitting process was repeated multiple times to ensure consistent results and test for lack of convergence.

The model was fitted to data from both cohorts and the parameter estimates are presented in Table S3. Prior distributions for the global parameters were derived from a study of the Phase 2 data[6]. Note that very informative priors were selected for the half-life of the short-lived component of the antibody response, due to the lack of measurements between the peak antibody response at month 3, and the next measurement at month 20 before the booster dose. Thus, the Phase 3 data has very little statistical power to estimate the half-life of the short-lived component of the antibody response.

Table S3: Estimates of parameters describing the dynamics of RTS,S-induced anti-CS antibodies using mixed effects models. Priors and posteriors are presented as median and 95% credible intervals. U denotes a uniform distribution. Gamma priors were assumed for d_s and d_l. Beta priors were assumed for ρ_{peak} and ρ_{boost}. Note that the mean and median of a distribution are not necessarily equal.

			**6-12 week category**	**5-17 month category**
	parameter	***prior***	***posterior***	
d_s	half-life of short-lived component of antibody response	46 (43, 49) days	45 (43, 48) days	45 (42, 48) days
d_l	half-life of long-lived component of antibody response	572 (269, 1045) days	634 (574, 709) days	591 (557, 632) days
ρ_{peak}	proportion of short-lived component following primary schedule	0·83 (0·63, 0·95)	0·93 (0·92, 0·94)	0·88 (0·87, 0·89)
ρ_{boost}	proportion of short-lived component following booster dose	0·83 (0·63, 0·95)	0·79 (0·77, 0·81)	0·70 (0·68, 0·72)
σ_s	standard deviation in half-life of short-lived antibody component	U(0, 5000) days	20 (14, 26) days	16 (11, 22) days
σ_l	standard deviation in half-life of long-lived antibody component	U(0, 5000) days	373 (305, 466) days	245 (209, 286) days
$\sigma_{\rho,peak}$	standard deviation in proportion short-lived antibodies after primary schedule	U(0, 5000)	0·08 (0·07, 0·09)	0·10 (0·09, 0·11)
$\sigma_{\rho,boost}$	standard deviation in proportion short-lived antibodies following booster dose	U(0, 5000)	0·19 (0·17, 0·21)	0·19 (0·17, 0·20)
σ_{obs}	observational variance (log-Normal)	U(0, 5000)	0·34 (0·33, 0·35)	0·35 (0·34, 0·36)

3.3. Alternative models of antibody dynamics

The mathematical model of antibody dynamics described above assumes that following vaccination, the decay of anti-CS antibody titres over time can be described by a bi-phasic exponential process. This corresponds to an immunological model where antibodies are generated by two populations of antibody secreting plasma B cells: one short-lived and one long-lived[20]. This model is equivalent to the plasma cell driven kinetic (PCDK) model described by Andraud *et al*[21]. The decay of vaccine-induced antibody responses has also been described using a power law model[22]. Here we describe details of two alternative models of antibody dynamics following vaccination: a single exponential decay model; and a power law decay model.

Under a single exponential decay model, following primary vaccination with RTS,S, it is assumed that anti-CS antibody titres decay exponentially with half-life d_{peak} as follows:

$$CS\left(t\right) = CS_{\text{peak}} e^{-r_{\text{peak}} t} \tag{S6}$$

where r_{peak} = log(2)/d_{peak}. Following a booster dose at time t_{boost} it is assumed that anti-CS antibody titres are boosted to CS_{boost} and decay exponentially with half-life d_{boost} as follows:

$$CS(t) = CS_{\text{boost}} e^{-r_{\text{boost}}\left(t - t_{\text{boost}}\right)} \tag{S7}$$

where r_{boost} = log(2)/d_{boost}.

Under a conventional power law decay model as described by Fraser *et al*[22], following primary vaccination with RTS,S it is assumed that anti-CS antibody titres are described by:

$$CS(t) = CS_{\text{peak}} \left(1 + \frac{r_{\text{peak}} t}{k_{PL}}\right)^{-k_{PL}} \tag{S8}$$

where k_{PL} is a shape parameter to be estimated. In particular, k_{PL} is the shape parameter of a Gamma distribution. The model assumes that antibodies are generated by antibody secreting cells which have half-lives distributed according to a Gamma distribution. Following a booster dose at time t_{boost} it is assumed that anti-CS antibody titres are boosted to CS_{boost} and decay as follows:

$$CS(t) = CS_{\text{boost}} \left(1 + \frac{r_{\text{boost}} \left(t - t_{\text{boost}}\right)}{k_{PL}}\right)^{-k_{PL}} \tag{S9}$$

Both models were fitted to the same data using the same methods as for the bi-phasic exponential described above. Table S4 shows the estimated parameters for the single phase exponential model. Table S5 shows the estimated parameters for the conventional power law decay model. Figure S11 shows a comparison between the single exponential model and the data. Visual examination of the model fits in Figure S11 is sufficient to demonstrate that the single exponential model does not replicate the patterns of decay of antibody response observed in the data.

Table S4: Estimates of parameters describing the dynamics of RTS,S-induced anti-CS antibodies using a single exponential model fitted using mixed effects methods. Priors and posteriors are presented as median and 95% credible intervals. U denotes a uniform distribution. Gamma priors were assumed for d_{peak} and d_{boost}. Note that the mean and median of a distribution are not necessarily equal.

			**6-12 week category**	**5-17 month category**
	parameter	***prior***	***posterior***	
d_{peak}	half-life of antibody response after primary vaccination	164 (20, 583) days	130 (127, 133) days	160 (157, 164) days
d_{boost}	half-life of antibody response after booster dose	164 (20, 583) days	136 (129, 144) days	193 (185, 204) days
σ_{peak}	standard deviation in half-life of antibodies after primary vaccination	U(0, 5000) days	43 (40, 46) days	56 (52, 60) days
σ_{boost}	standard deviation in half-life of antibodies after booster dose	U(0, 5000) days	64 (56, 73) days	86 (76, 97) days
σ_{obs}	observational variance (log-Normal)	U(0, 5000)	0·72 (0·70, 0·74)	0·66 (0·65, 0·68)

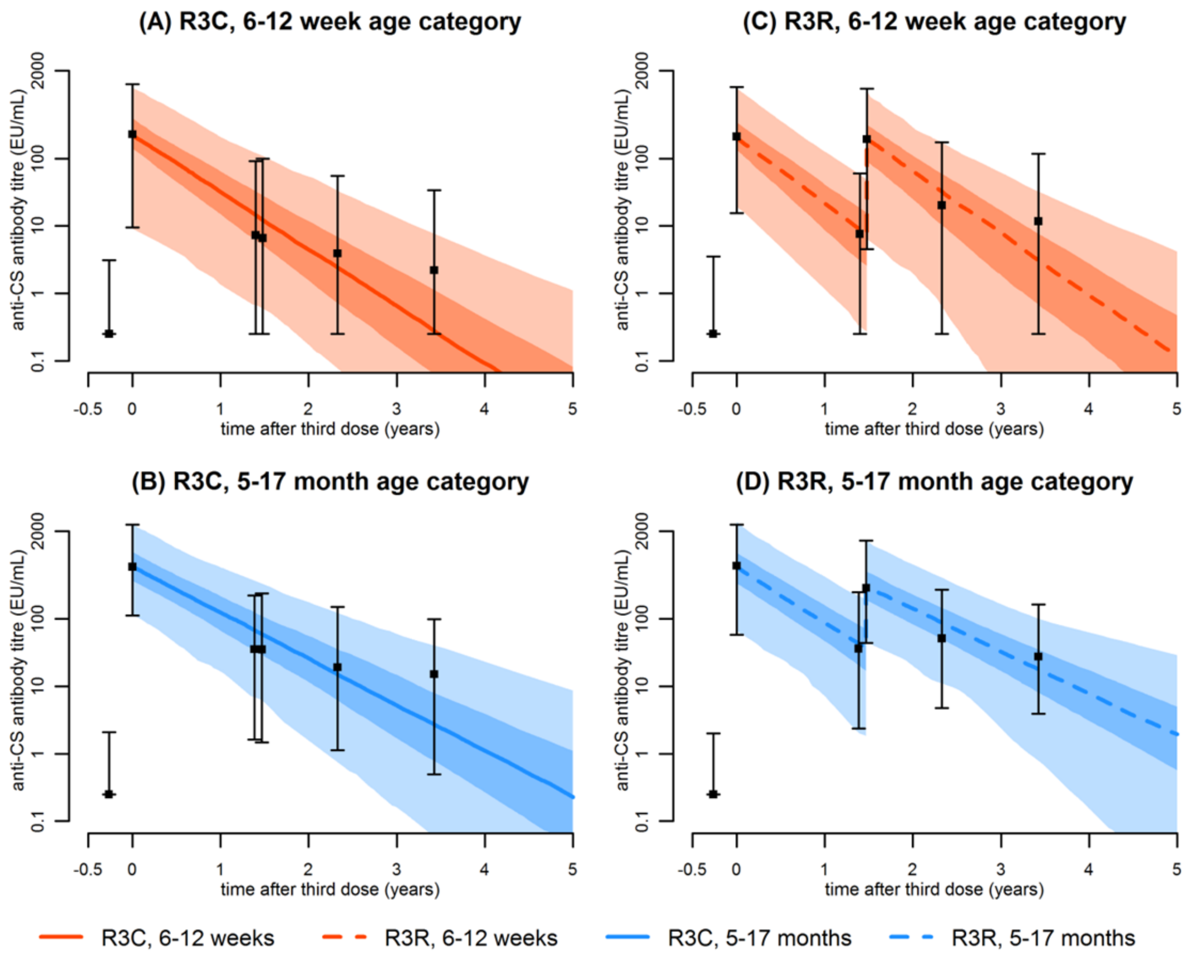

Figure S11: Comparison between single phase exponential model of antibody dynamics and measured anti-CS antibody titres with or without booster. The black bars denote the median and 95% ranges of the data. The solid and dashed curves denote the median of the model predicted antibody titres. The dark and light shaded regions represent 50% and 95% of the model predicted variation in antibody titres.

Table S5: Estimates of parameters describing the dynamics of RTS,S-induced anti-CS antibodies using a conventional power law decay model fitted using mixed effects methods. Priors and posteriors are presented as median and 95% credible intervals. U denotes a uniform distribution. Gamma priors were assumed for d_{peak}, d_{boost} and k_{PL}. Note that the mean and median of a distribution are not necessarily equal.

			6-12 week category	5-17 month category
	parameter	***prior***	***posterior***	
d_{peak}	half-life of antibody response after primary vaccination	164 (20, 583) days	49 (45, 54) days	57 (53, 61) days
d_{boost}	half-life of antibody response after booster dose	164 (20, 583) days	88 (78, 101) days	104 (95, 113) days
σ_{peak}	standard deviation in half-life of antibodies after primary vaccination	U(0, 5000) days	74 (65, 87) days	65 (58, 74) days
σ_{boost}	standard deviation in half-life of antibodies after booster dose	U(0, 5000) days	134 (109, 169) days	96 (83, 113) days
k_{PL}	shape parameter for conventional power law gamma distribution	4 (0.4, 15.3)	1.78 (1.75, 1.81)	1.60 (1.56, 1.63)
σ_{obs}	observational variance (log-Normal)	U(0, 5000)	0·36 (0·35, 0·37)	0·38 (0·37, 0·39)

4. Association between anti-CS antibodies and efficacy

4.1. Vaccine efficacy profiles

Measurement of the effectiveness of malaria vaccines in Phase 2 and Phase 3 trials has focussed on statistical estimation of point estimates of vaccine efficacy over fixed time periods (e.g. 0-9 months, 0-2 years)[23-26]. Although adequately powered to accurately assess efficacy, most conventional statistical methods are not designed to robustly estimate the duration of protection[27]. Here we describe statistical methods for estimating both efficacy and duration of protection. We define the vaccine efficacy profile to be the combination of initial efficacy and the pattern of decay over time. A vaccine efficacy profile can be considered for various endpoints, in particular *P. falciparum* infection and episodes of clinical malaria. Three different vaccine efficacy profiles are considered here.

4.1.1. Exponential profile

Vaccine efficacy is assumed to begin at V_0 and wane exponentially over time with rate $r_0 = \frac{\log(2)}{d_0}$ where d_0 is the half-life.

$$V(t) = V_0 e^{-r_0 t} \tag{S10}$$

After a booster dose at time t_{boost} vaccine efficacy will increase to V_{boost} and wane exponentially with rate $r_{\text{boost}} = \frac{\log(2)}{d_{\text{boost}}}$ where d_{boost} is the half-life. For $t > t_{\text{boost}}$ efficacy is given by the following equation:

$$V(t) = V_{\text{boost}} e^{-r_{\text{boost}}(t - t_{\text{boost}})} \tag{S11}$$

4.1.2. Bi-phasic exponential profile

Vaccine efficacy is assumed to begin at V_0 and wane over time according to a bi-phasic exponential pattern with decay rates $r_{\text{bp,s}}$ and $r_{\text{bp,l}}$. The proportion of initial vaccine efficacy that is short-lived is given by $\rho_{\text{bp,0}}$. Efficacy at time t after vaccination is as follows.

$$V(t) = V_0 \left(\rho_{\text{bp,0}} e^{-r_{bp,s} t} + \left(1 - \rho_{\text{bp,0}}\right) e^{-r_{bp,l} t} \right) \tag{S12}$$

After a booster dose at time t_{boost} efficacy is boosted to V_{boost}. The rates of decay of the short-lived and long-lived components of vaccine efficacy may change after the booster dose, but we assume they remain the same. However, we assume that the proportion of efficacy that is short-lived may change to $\rho_{\text{bp,boost}}$ after the booster dose. For $t > t_{\text{boost}}$ we have

$$V(t) = V_{\text{boost}} \left(\rho_{\text{bp,boost}} e^{-r_{bp,s}(t - t_{\text{boost}})} + \left(1 - \rho_{\text{bp,boost}}\right) e^{-r_{bp,l}(t - t_{\text{boost}})} \right) \tag{S13}$$

4.1.3. Antibody profile

We consider the special case where the waning of efficacy against infection over time can be determined by the waning of vaccine-induced antibody responses. In particular, we assume that following vaccination with RTS,S/AS01 the change in anti-CS antibody titres can be described by equations (S1) and (S2). The model-predicted antibody titre at time *t* can be used to predict vaccine efficacy against *P. falciparum* infection via a dose-response curve defined as follows:

$$V(t) = V_{\max}\left(1 - \frac{1}{1+\left(\frac{CS(t)}{\beta}\right)^{\alpha}}\right) \qquad \text{(S14)}$$

where $V_{\max}$, α and β are parameters to be estimated. Equation (S12) is a functional formal regularly used in pharmacokinetics/pharamacodynamics for modelling dose-relationships[28]. It is a flexible function (a Hill function) that can capture a range of behaviours (Figure S12). V_{max} determines the maximum possible vaccine efficacy. β is a scale parameter. When antibody titre = β, vaccine efficacy is at half its maximum level. The shape parameter α determines the slope of the curve. Large values of α result in a threshold antibody titre for protection. Small values of α result in a gradual increase in efficacy with increasing antibody titre.

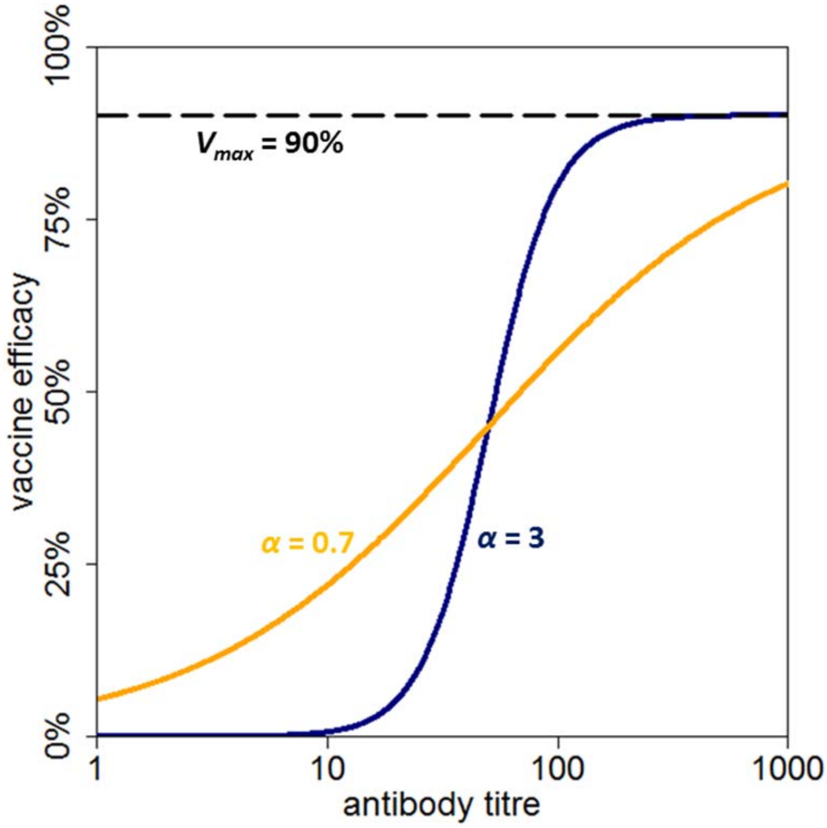

Figure S12: Examples of dose-response relationships described by equation (S14). It is assumed that β = 50 and V_{max} = 90%.

4.2. Seasonality in exposure

There is substantial seasonal variation in the incidence of clinical malaria in the control cohort in all sites (Figure S1). The following functional form has been used by Griffin *et al*[29] to capture patterns of seasonal malaria transmission with a single peak:

$$S(t) = S_0\left(c + (1-c)\left(\frac{1+\cos(\theta)}{2}\right)^{\kappa}\right) \quad \text{(S15)}$$

where $\theta = 2\pi\left(\frac{t}{365} - u\right)$. S_0 is a normalising constant defined such that $\int_0^{365} S(t)dt = 1$ which can be evaluated numerically, or analytically as described by Griffin[30].

Here we extend this functional form to also account for geographical regions with two distinct peaks in malaria transmission (e.g. parts of east Africa with two rainy seasons).

$$S(t) = S_0\left(c + \nu(1-c)\left(\frac{1+\cos(\theta_1)}{2}\right)^{\kappa_1} + (1-\nu)(1-c)\left(\frac{1+\cos(\theta_2)}{2}\right)^{\kappa_2}\right) \quad \text{(S16)}$$

where $\theta_1 = 2\pi\left(\frac{t}{365} - u_1\right)$ and $\theta_2 = 2\pi\left(\frac{t}{365} - u_2\right)$. Figure S13 shows the best fit of the double-peak seasonality profile in equation (S16) to data on episodes of clinical malaria from the control cohort for each of the 11 trial sites. The seasonality parameters for each site are provided in Table S6.

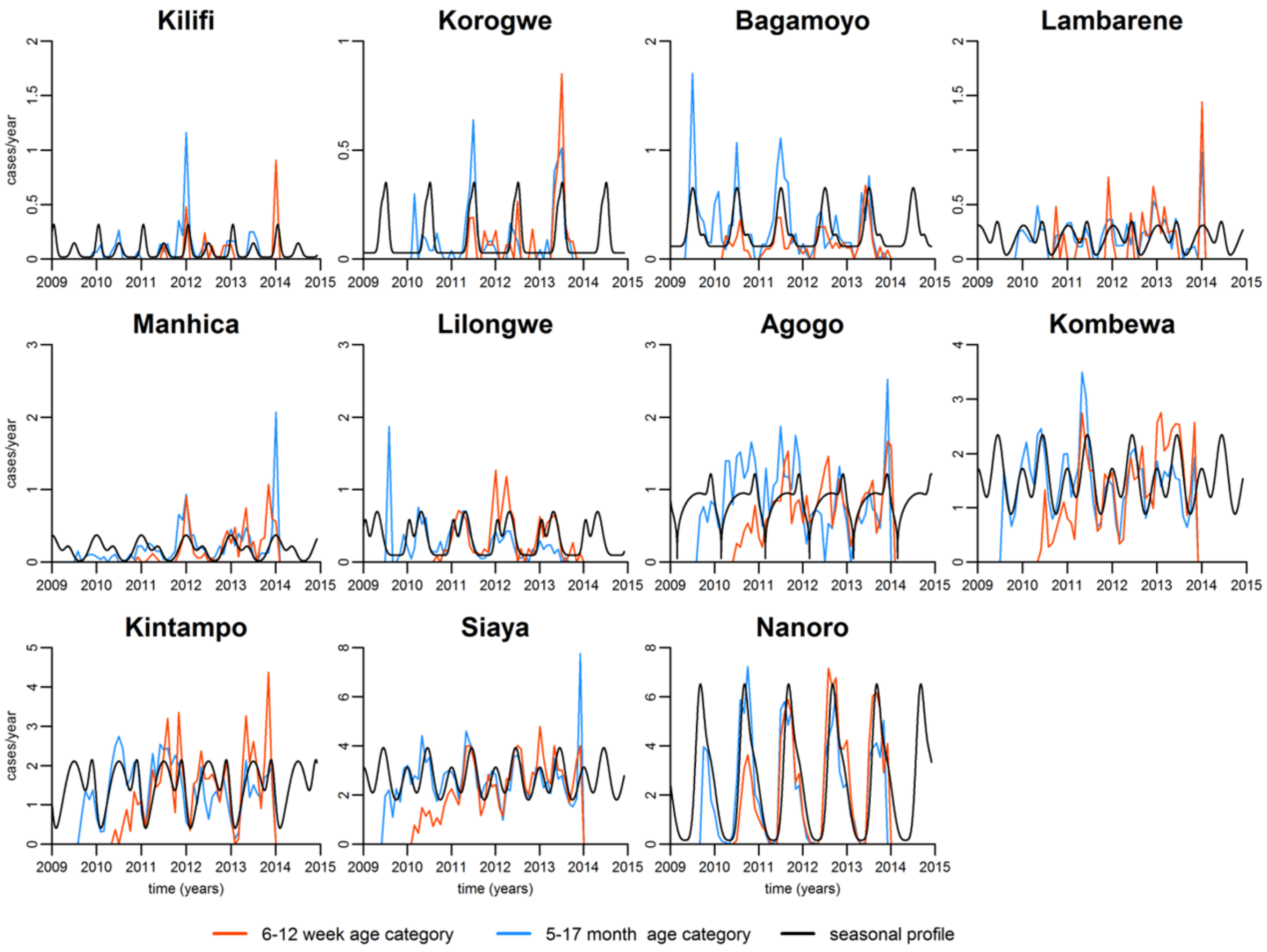

Figure S13: Seasonal patterns in incidence of clinical malaria (primary case definition) in the control cohort. The best-fit line of the seasonality profile with a double peak for each site is shown in black.

Table S6: Estimated parameters for seasonality profiles for each site. *c* was constrained in the region [0.02,1]. *v*, u_1 and u_2 were constrained in the region [0,1]. κ_1 and κ_2 were constrained in the region [0, 20].

Trial site	*c*	v	u_1	κ_1	u_2	κ_2	S_0
Kilifi	0·043	0·699	0·044	19·99	0·500	11·31	5·74
Korogwe	0·054	0·398	0·414	19·99	0·523	19·99	5·80
Bagamoyo	0·154	0·159	0·755	19·06	0·507	6·32	3·05
Lambarene	0·020	0·524	0·440	8·26	0·022	1·47	3·14
Manhica	0·020	0·361	0·366	5·90	0·999	2·47	3·18
Lilongwe	0·081	0·441	0·043	15·14	0·314	4·35	3·65
Agogo	0·038	0·703	0·651	0·162	0·919	19·78	1·58
Kombewa	0·212	0·383	1·000	3·28	0·447	2·91	2·19
Kintampo	0·020	0·479	0·911	7·67	0·499	0·94	2·67
Siaya	0·310	0·393	0·003	4·08	0·456	3·66	1·99
Nanoro	0·020	0·550	0·656	6·73	0·841	1·68	3·19

4.3. Models for the incidence of clinical malaria

The pattern of incidence of symptomatic episodes of clinical malaria will depend on exposure to infectious mosquito bites and age. Such patterns can be described by age-incidence curves. Age-incidence curves can be estimated statistically from epidemiological data on the incidence of clinical malaria[2, 31]. Alternatively they can be estimated using mathematical models that incorporate biological insights into the acquisition of clinical immunity[29, 32, 33]. The advantage of a statistical age-incidence model is that it allows estimation of clinical incidence in a trial site given an individual's age. However, age-incidence curves do not allow for robust comparisons between sites of differing transmission intensity. Mathematical models accounting for the age and exposure dependent acquisition of clinical immunity allow for estimates of the probability that blood-stage *P. falciparum* infection will progress to an episode of clinical malaria. Here, we describe the immunity acquisition model used in the main manuscript. In Section 5 we describe an alternative approach using age-incidence curves for the incidence of episodes of clinical malaria[2].

The immunity acquisition model is based on a previously validated model of malaria transmission dynamics and the age and exposure dependent acquisition of clinical immunity[29, 32]. We assume that each participant n is subjected to a fixed entomological inoculation rate (EIR) depending on their trial site, denoted EIR^n. A participant's exposure to infectious bites will depend on their age a. Young children are assumed to receive fewer mosquito bites than adults due to their smaller body sizes and tendency to spend more time indoors at night time[4, 5]. The rate at which a trial participant of age a is exposed to infectious bites is thus dependent on their age and trial site and can be modelled as:

$$\varepsilon^n(a) = EIR^n \underbrace{\left(1-\rho_a e^{-a/a_0}\right)}_{\text{age dependency}} \tag{S17}$$

For individuals sleeping under LLINs, the rate of exposure to infectious bites is adjusted by a further factor γ_{LLIN}:

$$\varepsilon^n(a) = \gamma_{\text{LLIN}} EIR^n \underbrace{\left(1-\rho_a e^{-a/a_0}\right)}_{\text{age dependency}} \tag{S18}$$

The probability that a bite from an infectious mosquito progresses to blood-stage infection in an unvaccinated participant can be described by the previously described functional form[32]:

$$b(t) = b_0 \left(b_1 + \frac{1-b_1}{1+\left(I_B(t)/I_{B0}\right)^{\kappa_B}} \right) \tag{S19}$$

where $I_B(t)$ is the time-dependent immunity against infection, and b_0, b_1, I_{B0} and κ_B are immune parameters defined in Table S7. The probability of infection per mosquito bite $b(t)$ is assumed to decrease with increasing levels of infection-blocking immunity $I_B(t)$, however this is assumed to be independent of RTS,S induced anti-CS antibody titres CS(t). Note that this natural infection-blocking immunity is estimated to have very little effect in these young cohorts[34] and hence is just included here for completeness. The time-dependent hazard of infection on an unvaccinated trial participant at time t after vaccination is then given by:

$$\Lambda^n(t) = S^n(t)\varepsilon^n(a+t)b(t) \tag{S20}$$

where $S^n(t)$ is the seasonality profile in equation (S16). And the time-dependent hazard of infection on a vaccinated trial participant is

$$\Lambda^n(t) = S^n(t)\varepsilon^n(a+t)b(t)\left(1-V(t)\right) \tag{S21}$$

Using a similar functional form we can calculate the probability that an infection leads to an episode of clinical malaria[32]

$$\phi(t) = \phi_0\left(\phi_1 + \frac{1-\phi_1}{1+\left(\left(I_{CA}(t)+I_{CM}(t)\right)/I_{C0}\right)^{\kappa_C}}\right) \tag{S22}$$

where $I_{CA}(t)$ is the time-dependent immunity against clinical malaria, $I_{CM}(t)$ the time-dependent maternal immunity against clinical malaria, and ϕ_0, ϕ_1, I_{C0} and κ_C are immune parameters defined in Table S7. The time dependent hazard of an episode of clinical malaria in an unvaccinated trial participant can be modelled as

$$H^n(t) = S^n(t)\varepsilon^n(a+t)b(t)\phi(t) \tag{S23}$$

And the time-dependent hazard of an episode of clinical malaria in a vaccinated trial participant is given by

$$H^n(t) = S^n(t)\varepsilon^n(a+t)b(t)\left(1-V(t)\right)\phi(t) \tag{S24}$$

The rate at which episodes of clinical malaria were detected in trials was adjusted by a further factor r_{CLIN} to account for differences in the definition of a case of clinical malaria between the data used to parameterise the model[32] and the primary case definition in the trial. Thus

$$H^n(t) = r_{\text{clin}}S^n(t)\varepsilon^n(a+t)b(t)\left(1-V(t)\right)\phi(t) \tag{S25}$$

The acquisition of immunity against infection (I_B) and clinical malaria (I_{CA}) is described by the following set of differential equations

$$\begin{aligned}\frac{\partial I_B}{\partial t}+\frac{\partial I_B}{\partial a} &= \frac{\varepsilon(t)}{\varepsilon(t)u_B+1}-\frac{I_B}{d_B}\\ \frac{\partial I_{CA}}{\partial t}+\frac{\partial I_{CA}}{\partial a} &= \frac{\Lambda(t)}{\Lambda(t)u_C+1}-\frac{I_{CA}}{d_C}\end{aligned} \tag{S26}$$

where u_B, d_B, u_C and d_C are immune parameters as defined in Table S7. Maternal clinical immunity I_{CM} is assumed to be at birth a proportion P_M of the acquired immunity of a 20 year old and to decay at rate $1/d_M$.

$$I_{CM}(a) = P_M I_{CA}(20)e^{-a/d_M} \tag{S27}$$

Table S7: Parameters describing the acquisition of natural immunity against *P. falciparum* infection and episodes of clinical malaria. All parameters are taken from Griffin *et al*[32]. Estimated parameters are presented with 95% credible intervals.

Parameter description	Symbol	Value	95% credible interval
**Age-dependent exposure**			
Age-dependent biting parameter	ρ_a	0·85	Fixed
Age-dependent parameter	a_0	8 years	Fixed
**Immunity reducing probability of infection**			
Probability with no immunity	b_0	0·590	(0·389, 0·845)
Maximum relative reduction	b_1	0·5	Fixed
Inverse of decay rate	d_B	10 years	Fixed
Scale parameter	I_{B0}	43·879	(20·1, 120)
Shape parameter	κ_B	2·155	(1·22, 2·93)
Duration in which immunity is not boosted	u_B	7·199 days	(2·63, 15·0) days
**Immunity reducing probability of clinical disease**			
Probability with no immunity	Φ_0	0·792	(0·548, 0·961)
Maximum relative reduction	Φ_1	0·0007	(0·00005, 0·0025)
Inverse of decay rate	d_C	30 years	Fixed
Scale parameter	I_{C0}	18·024	(11·9, 26·7)
Shape parameter	κ_C	2·369	(1·99, 2·86)
Duration in which immunity is not boosted	u_C	6·063 days	(2·82, 11·1) days
New-born immunity relative to mother's	P_M	0·774	(0·536, 0·981)
Inverse of decay rate of maternal immunity	d_M	67·695 days	(59·0, 79·4) days

4.4. Model likelihood for survival analysis

Using the incidence of clinical malaria $H(t)$ from either the age-incidence model or the immunity-acquisition model we can construct a model likelihood using survival analysis methods. We assume that, accounting for seasonality in transmission, EIR remains constant throughout the trial, e.g. there is no decline in transmission over the five years of the trial.

A participant n in a vaccine trial with clinical malaria as an endpoint will be followed up for time T^n and experience I^n episodes of clinical malaria at times $\{\tau_1^n,\ldots,\tau_{I^n}^n\}$. We estimate two sets of parameters - EIR and θ where the latter denotes the set of model parameters for vaccine efficacy profiles. The likelihood of these parameters given the data is:

$$L^n\left(EIR^n,\theta\middle|I^n,\tau^n\right)=\left(\prod_{i=1}^{I^n}H^n\left(\tau_i^n\right)e^{\int_{\tau_i^n}^{\tau_i^n+T_{\text{cen}}}H^n(t)dt}\right)e^{-\int_0^{T^n}H^n(t)dt} \tag{S28}$$

Note that we have incorporated a censoring period after each episode into the likelihood (T_{cen} = 14 days). This accounts for the period of time after an episode of clinical malaria where a participant is under chemoprophylaxis

and thus protected from a second episode. In instances where two episodes of clinical are observed within T_{cen} = 14 days of each other in the data, the second episode is censored.

We can incorporate heterogeneity in exposure to infectious bites using a Gamma distribution with shape parameter k so that the mean number of infectious bites per day is EIR^n with variance $(EIR^n)^2/k$. Heterogeneity in exposure can be accounted for by integrating over x - the range of EIR with mean EIR^n and shape parameter k:

$$L^n\left(EIR^n, \mathrm{k}, \theta \middle| I^n, \tau^n\right) = \int_0^\infty \left(\left(\prod_{i=1}^{I^n} H^n\left(\tau_i^n\right) e^{\int_{\tau_i^n}^{\tau_i^n + T_{\text{cen}}} H^n(t)dt} \right) e^{-\int_0^{T^n} H^n(t)dt} \right) \Gamma_d\left(x \middle| EIR^n, k\right) dx \qquad \text{(S29)}$$

where $\Gamma_d(x|EIR^n,k)$ is a Gamma distribution with mean EIR^n and shape parameter k defined as follows:

$$\Gamma_d\left(x \middle| EIR^n, k\right) = \left(\frac{k}{EIR^n}\right)^k \frac{x^{k-1} e^{-\frac{kx}{EIR^n}}}{\Gamma(k)} \qquad \text{(S30)}$$

Note that the subscript d denotes the Gamma distribution as opposed to the Gamma function. Equation (S29) can be evaluated numerically using Gaussian quadrature with weights (w_j) and abscissas (x_j) derived from Gauss-Laguerre polynomials. We use 10 abscissas for numerical evaluation. The total likelihood for all N participants with episodes of clinical malaria as an endpoint can be obtained by multiplying each participant's likelihood in equation (S29):

$$L = \prod_{n=1}^{N} L^n \qquad \text{(S31)}$$

In the model likelihood described above episodes of clinical malaria are assumed to be due to variation in expsoure, age, LLIN status, an individual's level of naturally-acquired immunity, and an individual's vaccination status. After accounting for these factors, episodes of clinical malaria within an individual are assumed to be independent.

The model described above was fitted to data using Bayesian MCMC methods. The MCMC algorithm was implemented in C++. All updates were attempted with Normal proposal distributions. All Markov chains were visually examined for appropriate mixing and convergence. The MCMC fitting process was repeated twice to ensure consistent results and test for lack of convergence. Convergence was also formally tested for using the Gelman-Rubin statistic[18]. Posterior median parameter estimates with 95% credible intervals are presented in Table S8 for several vaccine efficacy profiles.

Table S8: MCMC parameter estimates for exponential, bi-phasic exponential and antibody models. EIR is presented in units of infectious bites per person per year. Half-life is presented in units of years. U denotes a uniform distribution. Log-Normal distributions were assumed for EIRs. Gamma priors were assumed for k, r_{clin}, γ_{LLIN}, d_{half}, d_{boost}, $d_{bp,s}$, $d_{bp,l}$, β_{CS} and α_{CS}. Beta priors were assumed for ρ_0, ρ_{boost} and V_{max}. Priors and posteriors are presented as median and 95% credible intervals. Note that the mean and median of a distribution are not necessarily equal.

		exponential	bi-phasic exponential	antibody	antibody (2 β)
Parameter	***Prior***		***Posterior***		
EIR: Kilifi	0·08 (0·03, 0·22)	0·25 (0·21, 0·30)	0·27 (0·22, 0·32)	0·39 (0·31, 0·48)	0·39 (0·32, 0·48)
EIR: Korogwe	0·15 (0·05, 0·39)	0·38 (0·33, 0·43)	0·40 (0·35, 0·46)	0·63 (0·52, 0·75)	0·63 (0·54, 0·76)
EIR: Bagamoyo	0·41 (0·15, 1·09)	0·85 (0·77, 0·95)	0·90 (0·82, 1·00)	1·31 (1·13, 1·51)	1·32 (1·15, 1·51)
EIR: Lambarene	0·29 (0·11, 0·76)	0·90 (0·79, 1·02)	0·95 (0·85, 1·09)	1·17 (0·99, 1·38)	1·18 (1·00, 1·39)
EIR: Manhica	3·67 (1·38, 9·77)	1·11 (0·94, 1·28)	1·14 (0·98, 1·33)	1·70 (1·38, 2·08)	1·75 (1·44, 2·13)
EIR: Lilongwe	0·92 (0·35, 2·45)	1·49 (1·35, 1·65)	1·56 (1·42, 1·71)	2·24 (1·92, 2·57)	2·28 (2·01, 2·61)
EIR: Agogo	3·72 (1·40, 9·91)	3·9 (3·6, 4·3)	4·1 (3·8, 4·5)	5·4 (4·7, 6·2)	5·5 (4·9, 6·2)
EIR: Kombewa	12·7 (4·8, 33·7)	8·8 (8·1, 9·7)	9·4 (8·6, 10·2)	14·8 (12·9, 17·0)	15·0 (13·4, 17·2)
EIR: Kintampo	4·91 (1·84, 13·08)	8·1 (7·4, 8·8)	8·6 (7·9, 9·4)	12·2 (10·7, 13·7)	12·4 (10·9, 13·7)
EIR: Siaya	12·8 (4·8, 34·1)	18·3 (17·0, 19·8)	19·4 (179, 20·6)	27·2 (23·8, 30·9)	27·6 (24·8, 30·8)
EIR: Nanoro	44·9 (16·8, 119·6)	17·6 (16·1, 19·1)	18·8 (172, 20·6)	27·7 (23·8, 32·3)	28·2 (24·6, 32·0)
k (Γ heterogeneity)	0·44 (0·09, 1·24)	1·09 (1·05, 1·14)	1·10 (1·06, 1·15)	1·16 (1·10, 1·23)	1·16 (1·09, 1·22)
r_{clin}	0·93 (0·32, 2·05)	1·48 (1·43, 1·54)	1·50 (1·45, 1·56)	1·36 (1·31, 1·42)	1·35 (1·30, 1·40)
γ_{LLIN}	U(0, 10)	0·86 (0·81, 0·91)	0·85 (0·80, 0·91)	0·70 (0·64, 0·77)	0·69 (0·64, 0·75)
β (6-12 weeks)	24·5 (1·4, 112·3)			97·6 (66·5, 134·1)	66·6 (48·7, 95·4)
β (5-17 months)	24·5 (1·4, 112·3)			97·6 (66·5, 134·1)	112·6 (80·7, 152·2)
α	0·92 (0·27, 2·19)			0·75 (0·63, 0·93)	0·82 (0·67, 1·02)
V_{max}	0·91 (0·74, 0·99)			0·93 (0·82, 0·99)	0·91 (0·82, 0·98)
V_0 (6-12 weeks)	U(0, 1)	0·74 (0·65, 0·82)	0·75 (0·67, 0·82)		
V_{boost} (6-12 weeks)	U(0, 1)	0·33 (0·21, 0·52)	0·46 (0·36, 0·55)		
d_0 (6-12 weeks)	1·84 (0·54, 4·38)	0·25 (0·21, 0·33)			
d_{boost} (6-12 weeks)	1·84 (0·54, 4·38)	0·60 (0·21, 2·04)			
$d_{bp,s}$ (6-12 weeks)	0·44 (0·09, 1·24)		0·24 (0·19, 0·30)		
$d_{bp,l}$ (6-12 weeks)	1·96 (1·14, 3·09)		2·16 (1·35, 3·23)		
$\rho_{bp,0}$ (6-12 weeks)	0·81 (0·60, 0·94)		0·91 (0·83, 0·97)		
$\rho_{bp,boost}$ (6-12 weeks)	0·81 (0·60, 0·94)		0·71 (0·56, 0·85)		
V_0 (5-17 months)	U(0, 1)	0·82 (0·77, 0·87)	0·87 (0·82, 0·91)		
V_{boost} (5-17 months)	U(0, 1)	0·43 (0·39, 0·48)	0·56 (0·52, 0·60)		
d_0 (5-17 months)	1·84 (0·54, 4·38)	0·37 (0·32, 0·44)			
d_{boost} (5-17 months)	1·84 (0·54, 4·38)	4·2 (2·9, 6·7)			
$d_{bp,s}$ (5-17 months)	0·44 (0·09, 1·24)		0·24 (0·20, 0·29)		
$d_{bp,l}$ (5-17 months)	1·96 (1·14, 3·09)		5·36 (4·27, 6·78)		
$\rho_{bp,0}$ (5-17 months)	0·81 (0·60, 0·94)		0·69 (0·63, 0·74)		
$\rho_{bp,boost}$ (5-17 months)	0·81 (0·60, 0·94)		0·19 (0·13, 0·27)		

4.5. Additional results

Here we compare the vaccine efficacy profiles for infection described in Section 4.1. In addition we consider a scenario where the antibody dose-response relationship in equation (S12) has different scale parameters for infants and children. The parameter estimates for each of the tested vaccine efficacy profiles are presented in Table S7.

The model with a separate dose-response curves for each age category (antibody model: 2β) was implemented by allowing a separate dose-response scale parameter for each age category: β_{6w12w} and β_{5m17m}. This model produced very similar dose-response curves to the antibody model with a single dose-response curve (Figure S14). In particular, the estimated curves from antibody model: 2β fell within the 95% credible intervals of the dose-response curve from the antibody model.

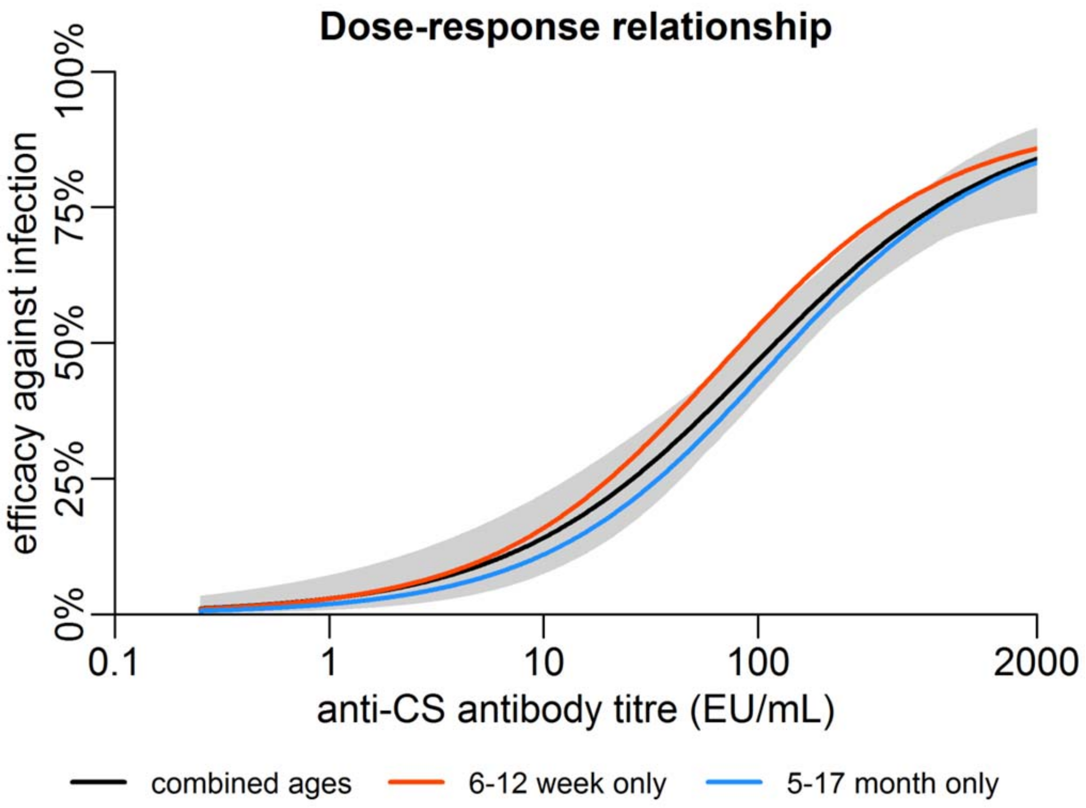

Figure S14: Comparison of the estimated dose-response curve obtained by fitting a single scale parameter β to all participants (antibody model), with the estimated dose-response curves obtained by fitting separate scale parameters to each of the age categories (antibody model (2β) in the notation of Table S7). The shaded grey region denotes the 95% credible interval for the estimated dose-response curve for the antibody model fitted to data from all participants

Figure S15 shows a comparison of the vaccine efficacy profile for infection for the antibody model and the exponential and bi-phasic exponential models. The bi-phasic and antibody vaccine efficacy profiles predict similar efficacy against infection. The exponential efficacy profile is a poor model choice, as efficacy against infection wanes to zero after approximately two years, long before efficacy against clinical malaria wanes to zero (Figure 2 and Figure 3). In both age categories, the exponential model matches the short-lived component of the bi-phasic exponential profile resulting in efficacy waning to zero after two years.

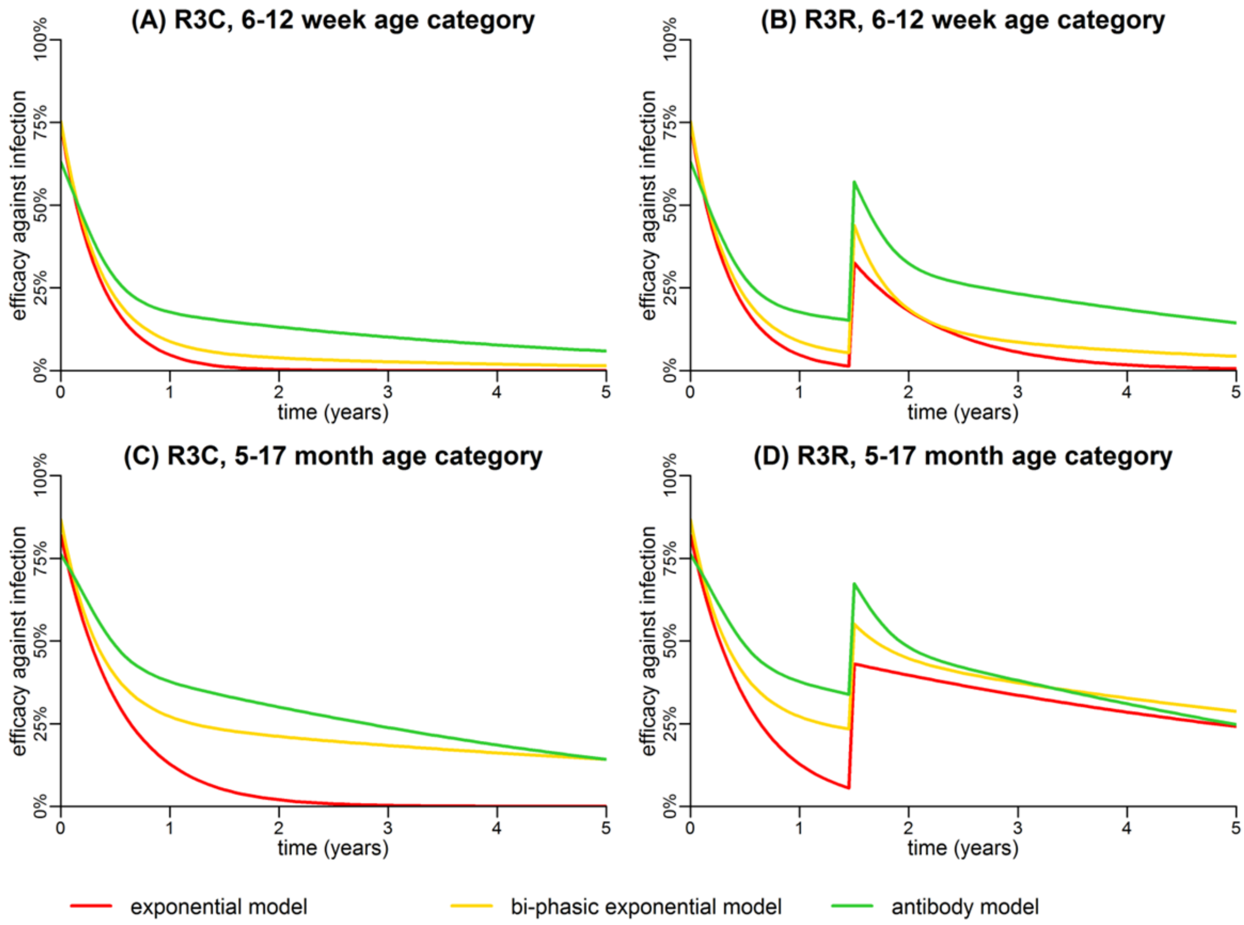

Figure S15: Comparison of predicted efficacy against infection for the exponential, bi-phasic exponential and antibody models.

Given the efficacy profile for infection the efficacy profile for clinical malaria can also be predicted, however it will be dependent on transmission intensity. Figures S16 show the estimated efficacy against clinical malaria at a range of transmission intensities (EIR = 1, 5, 10, 30, 50) using the antibody model. Efficacy against clinical malaria decays more rapidly than efficacy against infection (especially in high transmission settings) due to higher levels of natural immunity in the control cohort than the vaccine cohort. In high transmission settings incidence of clinical malaria in the vaccine cohort may be greater than incidence in the control cohort after 3 – 4 years leading to negative efficacy. This is due to reduced rates of acquisition of immunity and the shifting of episodes of malaria from younger children to older ones.

Note that at very high transmission intensity, efficacy against clinical malaria is predicted to be negative, i.e. the incidence of clinical malaria is higher in the vaccine cohort than the control cohort at that time. In all cases tested here, the net number of cases averted by vaccination remains positive.

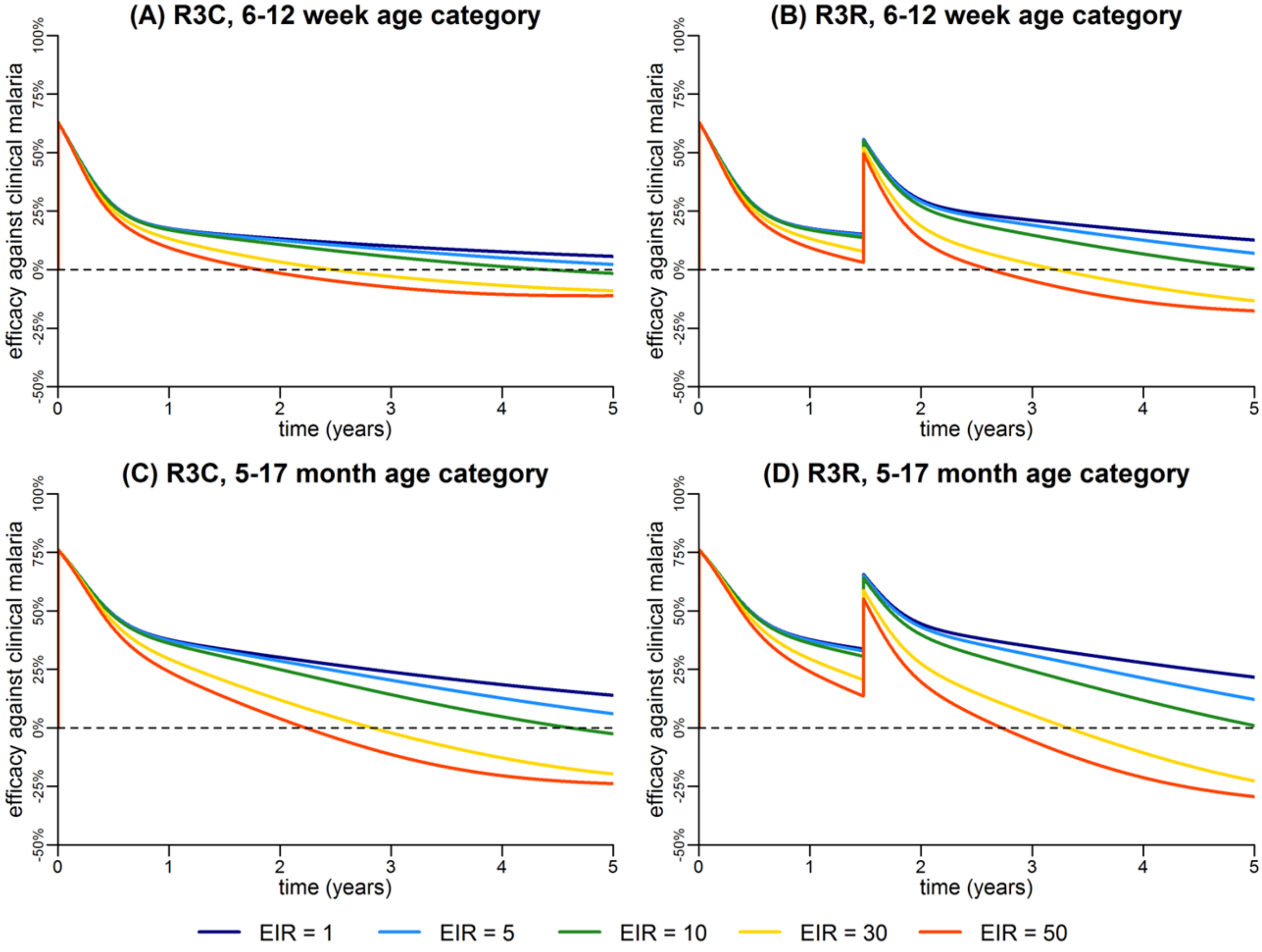

Figure S16: Comparison of predicted efficacy against clinical malaria for a range of transmission intensities.

4.5. Additional model validation

Figure S17 and Figure S18 show detailed model validation for Lilongwe and Nanoro, demonstrating how the model recreates seasonality and age-incidence patterns for each of the vaccine cohorts and both age categories. Similar plots are possible for the other nine trial sites.

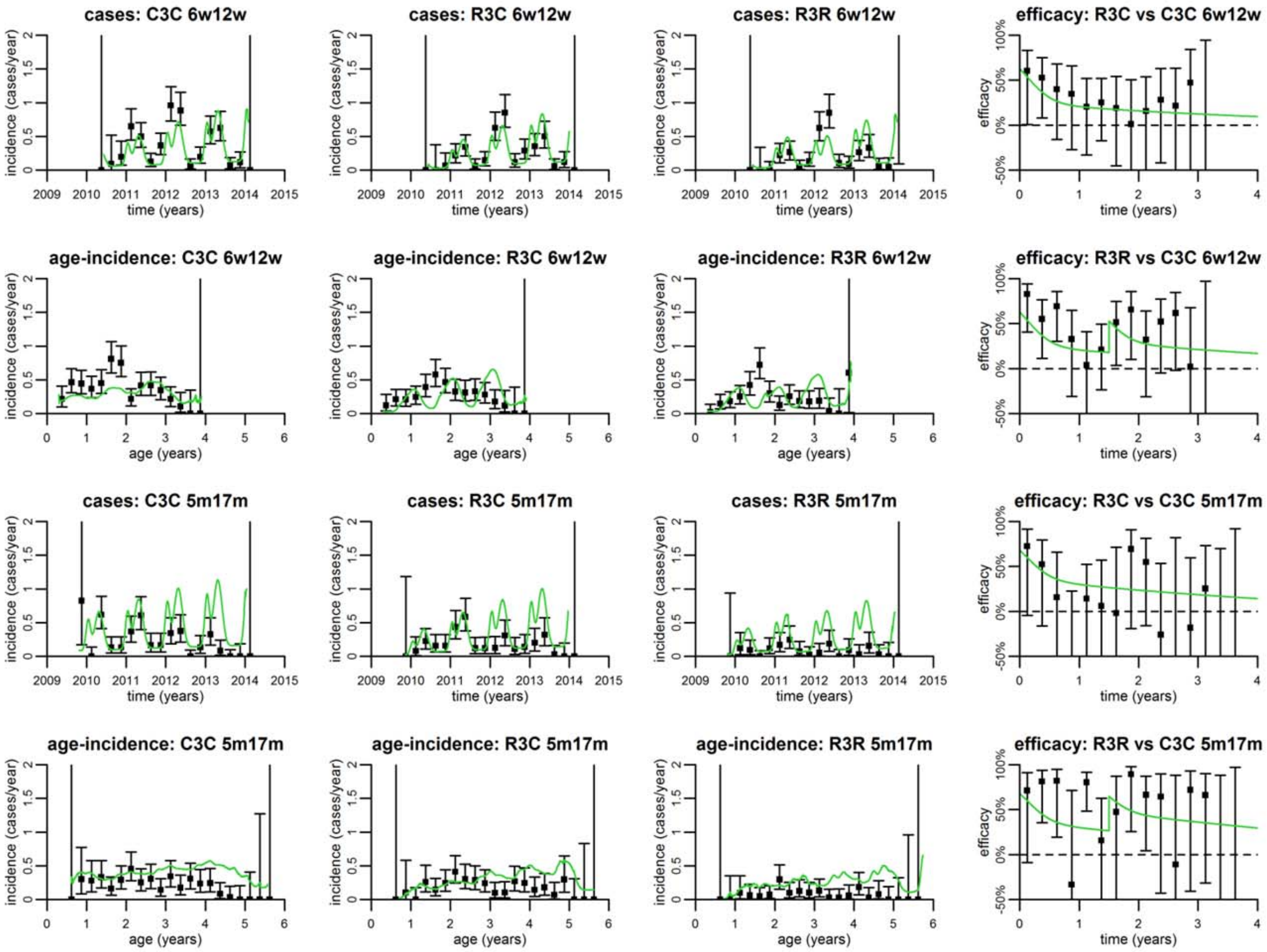

Figure S17: Validation of the antibody dynamics model for Lilongwe (a moderate transmission site).
The vaccine efficacy profile for infection is determined by anti-CS antibody titres and the dose-response relationship. The data are shown as cases over calendar time to present seasonal and temporal variation. Age-incidence curves are shown to present variation in incidence of clinical malaria with age. The right-hand column shows estimated efficacy against clinical malaria. Data are presented as point estimates with 95% confidence intervals. The posterior median of the age-incidence model is shown as a smooth line. 6w12w = 6-12 week age category. 5m17m = 5-17 month age category. C3C = control cohort. R3C = primary schedule of RTS,S/AS01 without a booster dose. R3R = primary schedule of RTS,S/AS01 with a booster dose.

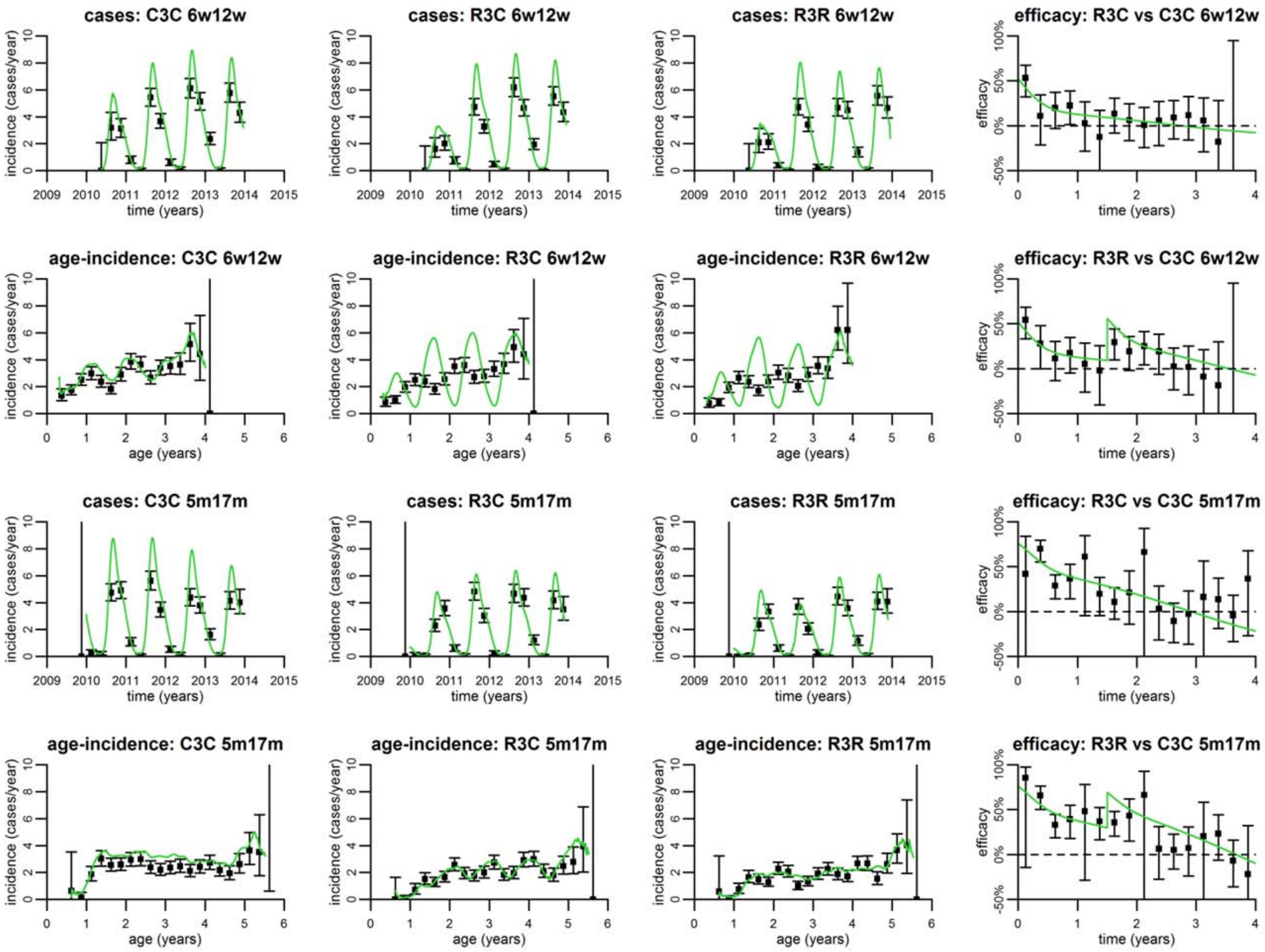

Figure S18: Validation of the antibody dynamics model for Nanoro (a high transmission site).
The vaccine efficacy profile for infection is determined by anti-CS antibody titres and the dose-response relationship. The data are shown as cases over calendar time to present seasonal and temporal variation. Age-incidence curves are shown to present variation in incidence of clinical malaria with age. The right-hand column shows estimated efficacy against clinical malaria. Data are presented as point estimates with 95% confidence intervals. The posterior median of the age-incidence model is shown as a smooth line. 6w12w = 6-12 week age category. 5m17m = 5-17 month age category. C3C = control cohort. R3C = primary schedule of RTS,S/AS01 without a booster dose. R3R = primary schedule of RTS,S/AS01 with a booster dose. Note the mismatch in seasonality patterns in the age-incidence curves for the 6-12 week age category. This is because the serological cohort was a subset of the total cohort. In particular, the first children enrolled were assigned to the serology cohort resulting in a different seasonal profile.

5. Age-incidence model

The incidence of clinical malaria depends on age and transmission intensity, amongst other factors[2, 31]. Carneiro *et al*[2] undertook a systematic review of studies of the dependence of clinical incidence on age, and found the age-incidence relationship to be well described by a log-Normal distribution. We fit these age-incidence curves to data on cases of clinical malaria for each trial site, adjusting for seasonality and bed net usage.

An advantage of age-incidence models is that they do not depend on the assumptions inherent in mathematical models of malaria transmission or the acquisition of natural immunity[32, 35, 36]. Statistical age-incidence models can be fitted to data from one trial site at a time. Data from multiple sites with different transmission intensity cannot be combined using this method. Due to variation in transmission intensity, a separate estimate of the vaccine efficacy profile for clinical malaria is estimated for each trial site. A drawback of only being able to analyse data from a single trial site at a time is the limitation on statistical power, particularly in low transmission sites where no clear pattern of vaccine efficacy profile is evident from the data. Notably, we only fitted an exponential vaccine efficacy profile. The data were not informative enough to justify the additional parameters required for more detailed profiles such as a bi-phasic exponential efficacy profile.

5.1. Model description

Following the approach of Carneiro *et al*[2] we assume that for each trial site, the relationship between age and the incidence of clinical malaria can be described by a log-Normal distribution as follows:

$$H(a) = H_0 \frac{e^{-\frac{(\log(a)-\mu_{LN})^2}{2\sigma_{LN}^2}}}{a\sigma_{LN}\sqrt{2\pi}} \tag{S32}$$

H_0, μ_{LN} and σ_{LN} are parameters to be estimated for each trial site. If an individual is sleeping under a long-lasting insecticidal net (LLIN) then the incidence of clinical malaria is assumed to be adjusted by a factor γ_{LLIN} such that

$$H_{\text{LLIN}}(a) = \gamma_{\text{LLIN}} H_0 \frac{e^{-\frac{(\log(a)-\mu_{LN})^2}{2\sigma_{LN}^2}}}{a\sigma_{LN}\sqrt{2\pi}} \tag{S33}$$

Vaccination is assumed to reduce the incidence of clinical malaria by a factor 1 – $V(t)$ in vaccinated individuals. A vaccine efficacy profile for clinical malaria with exponentially waning efficacy was assumed.

$$V(t) = V_0 e^{-r_0 t} \tag{S34}$$

where V_0 is the initial efficacy against clinical malaria at the start of follow-up, $r_0 = \log(2)/d_0$ is the rate of decay and d_0 is the half-life. A booster dose at time t_{boost} is assumed to increase vaccine efficacy to V_{boost} with decay rate $r_{\text{boost}} = \log(2)/d_{\text{boost}}$:

$$V(t) = V_{\text{boost}} e^{-r_{\text{boost}}(t-t_{\text{boost}})} \tag{S35}$$

5.2. Results of age-incidence model

The age-incidence model was fitted separately to data from each of the 11 trial sites assuming an exponential vaccine efficacy profile for clinical malaria. The likelihood in equation (S31) was used to describe the fit of the model to the data and the same Bayesian MCMC fitting methods were used. Full posterior parameter estimates are provided in Table S9. The predicted efficacy profiles for clinical malaria are shown for the 6-12 week age category (Figure S19) and the 5-17 month age category (Figure S20).

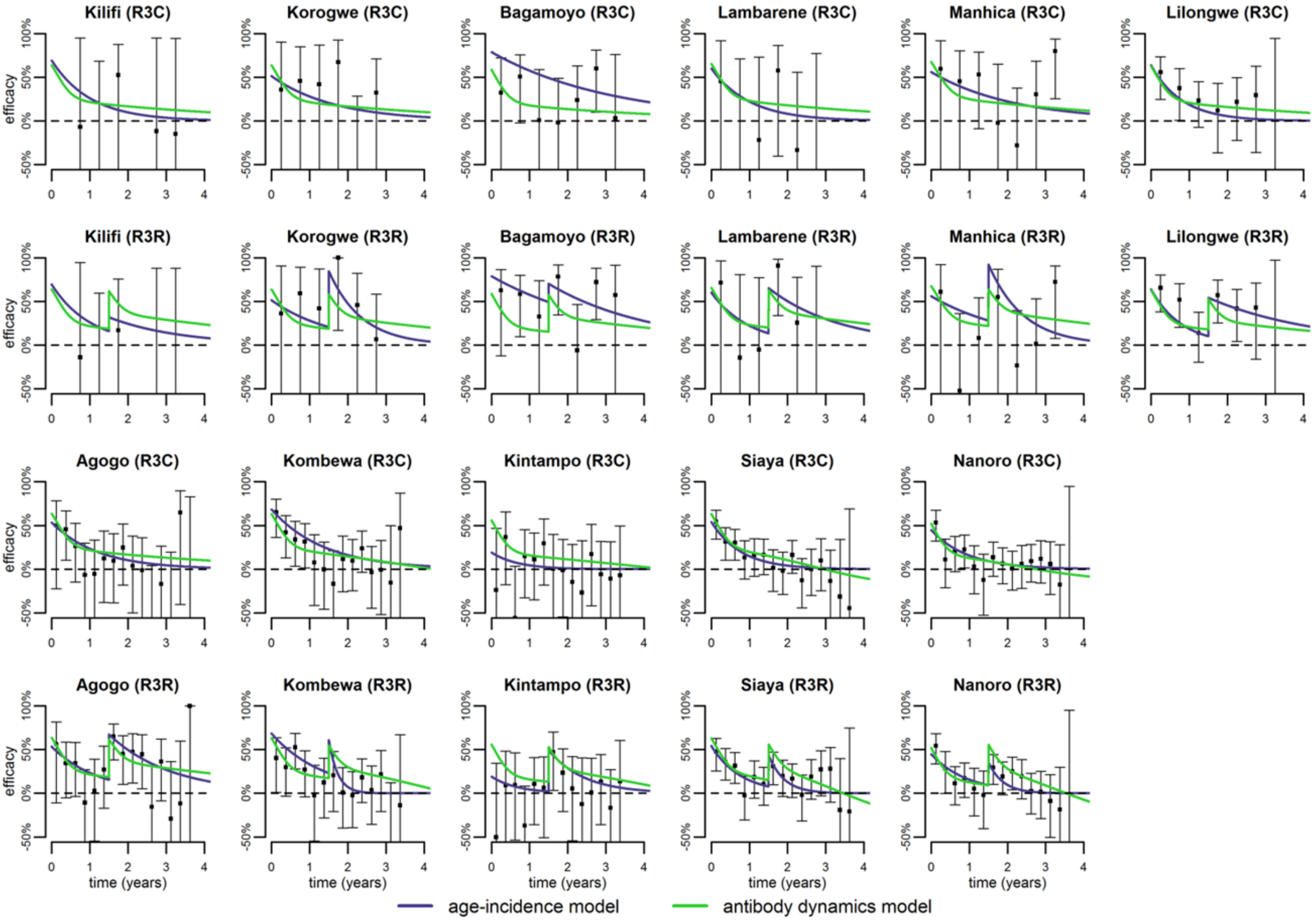

Figure S19: Vaccine efficacy profile for clinical malaria in the 6-12 week age category.
Data are presented as point estimates of efficacy against clinical malaria in 3 or 6 month windows with 95% confidence intervals. Cases of malaria are based on the primary case definition in the ATP population over the period M2·5 to SE. The posterior median estimates of efficacy against clinical malaria predicted by the age-incidence model and the antibody dynamics model are presented.

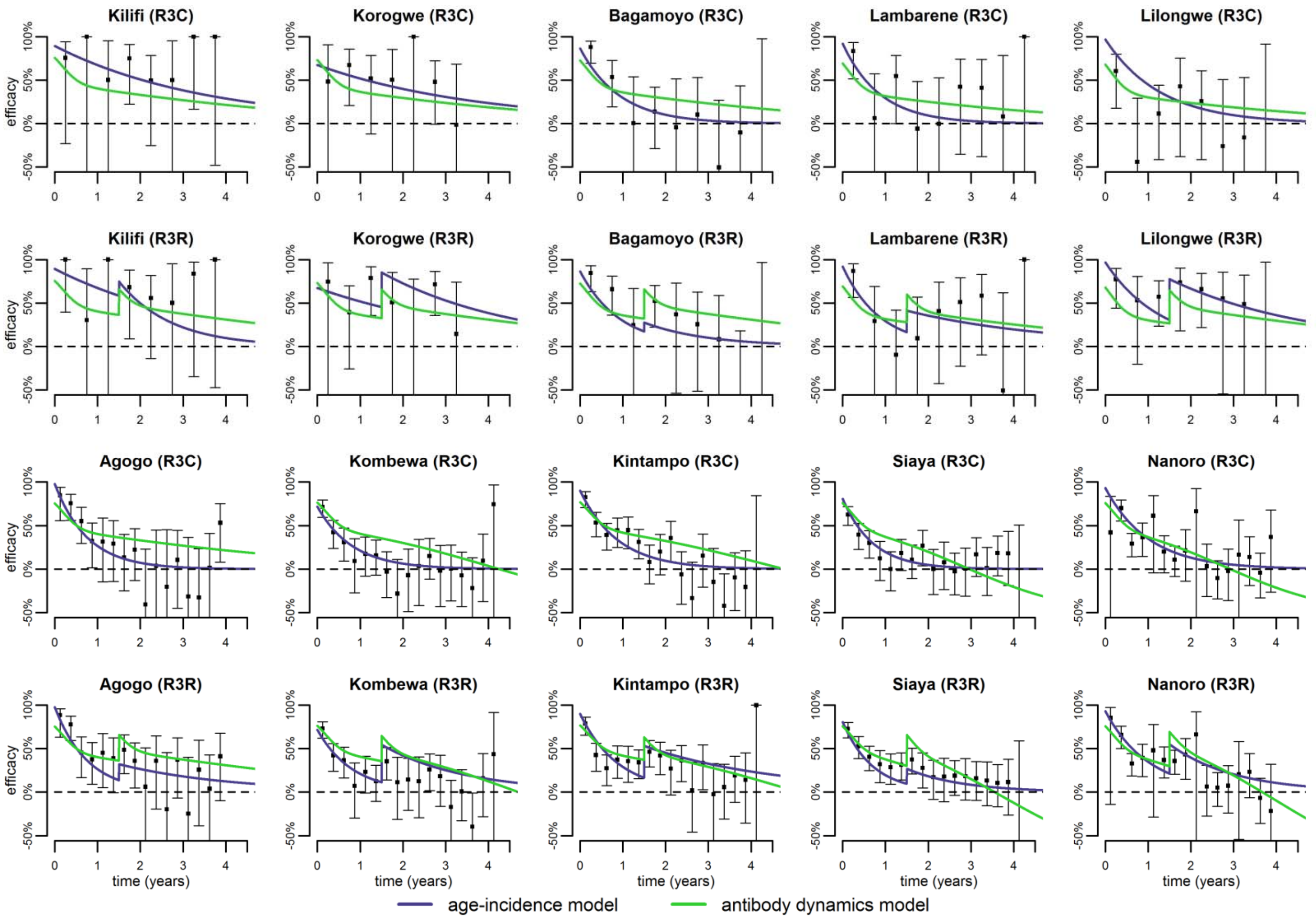

Figure S20: Vaccine efficacy profile for clinical malaria in the 5-17 month age category.
Data are presented as point estimates of efficacy against clinical malaria in 3 or 6 month windows with 95% confidence intervals. Cases of malaria are based on the primary case definition in the ATP population over the period M2·5 to SE. The posterior median estimates of efficacy against clinical malaria predicted by the age-incidence model and the antibody dynamics model are presented.

The results of fitting the age-incidence curves described by Carneiro *et al*[2] with an exponential vaccine efficacy profile for clinical malaria in Figure S18. In the low transmission sites there was limited statistical power to estimate initial efficacy and half-life of efficacy, resulting in wide credible intervals.

No significant association between initial efficacy V_0 and transmission intensity was observed (Figure S21A,E). In the 5-17 month age category, half-life of efficacy decreased with transmission intensity (Figure S21F). For example, in high transmission Siaya the half-life of efficacy was estimated as d_0 = 0.48 (95% CrI: 0.37, 0.66) years. In moderate transmission Lilongwe it was estimated as d_0 = 0.89 (95% CrI: 0.57, 1.41) years, and in low transmission Kilifi, d_0 = 2.45 (95% CrI: 0.81, 5.38) years. These results are consistent with more rapid waning of efficacy against clinical malaria in high transmission sites due to higher levels of naturally acquired immunity in the control cohort[25].

Following a booster dose in the 5-17 month age category the half-life of efficacy was estimated to increase in most trial sites. For example d_{boost} = 0.89 (95% Cr: 0.30, 2.65) years in Siaya, d_{boost} = 2.27 (95% CrI: 0.82, 6.26) years in Lilongwe, and d_{boost} = 0.85 (0.07, 5.07) years in Kilifi. However, due to smaller sample sizes in the R3R cohort and the

shorter duration of follow-up after the booster dose, there was limited statistical power to estimate the duration of protection of the booster dose.

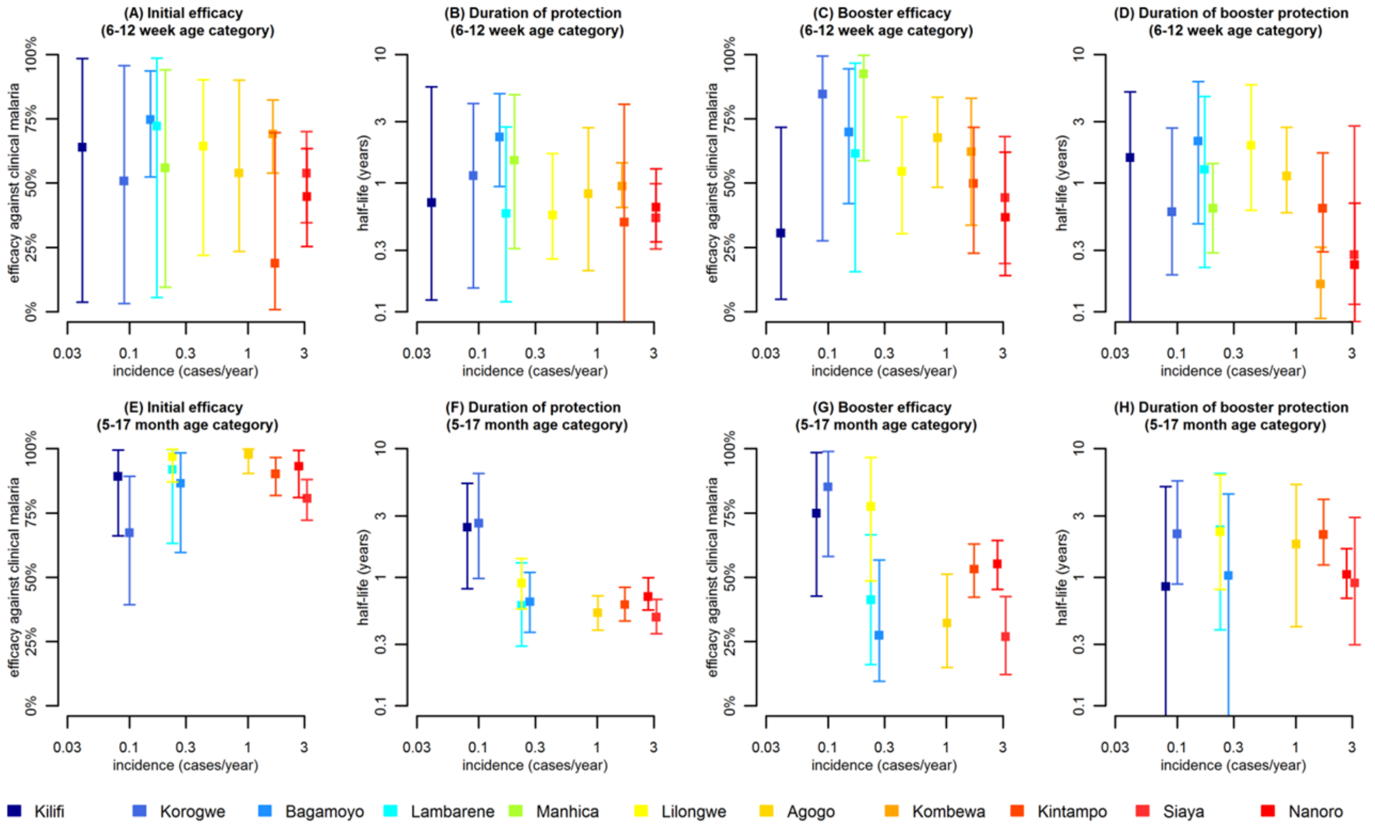

Figure S21: Estimates of initial efficacy against clinical malaria at the start of follow-up and duration of protection. Estimates are based on statistical age-incidence models with an exponential vaccine efficacy profile for clinical malaria (primary case definition). **(A)** Initial efficacy following primary schedule of RTS,S/AS01 in the 6-12 week age category. **(B)** Half-life of efficacy following primary schedule of RTS,S/AS01 in the 6-12 week age category. **(C)** Efficacy following a booster dose of RTS,S/AS01 in the 6-12 week age category. **(D)** Half-life of efficacy following a booster dose of RTS,S/AS01 in the 6-12 week age category. **(E)** Initial efficacy following primary schedule of RTS,S/AS01 in the 5-17 month age category. **(F)** Half-life of efficacy following primary schedule of RTS,S/AS01 in the 5-17 month age category. **(G)** Efficacy following a booster dose of RTS,S/AS01 in the 5-17 month age category. **(H)** Half-life of efficacy following a booster dose of RTS,S/AS01 in the 5-17 week age category. Estimates are presented as posterior medians with 95% credible intervals (Table S6).

Table S9: MCMC parameter estimates for age-incidence models of clinical malaria[2] fitted to data from one trial site at a time. Half-life is presented in years.

		Kilifi	Korogwe	Bagamoyo	Lambarene	Manhica	Lilongwe
Parameter	***Prior***			***Posterior***			
H_0	U(0, 100)	0·100 (0·001, 0·195)	0·012 (0·004, 0·086)	0·075 (0·007, 0·193)	0·060 (0·007, 0·745)	0·033 (0·014, 0·056)	0·003 (0·002, 0·005)
μ_{LN}	U(0, 100)	7·53 (1·51, 9·53)	3·04 (2·20, 5·60)	5·99 (2·31, 8·06)	4·43 (1·85, 8·99)	2·60 (2·03, 3·08)	0·90 (0·77, 1·08)
σ_{LN}	U(0, 100)	2·47 (0·72, 3·09)	1·18 (0·91, 1·97)	2·32 (1·32, 2·93)	1·70 (0·92, 2·78)	0·96 (0·80, 1·16)	0·77 (0·68, 0·89)
k (Γ heterogeneity)	U(0, 100)	0·27 (0·16, 0·52)	0·33 (0·23, 0·48)	0·27 (0·22, 0·31)	0·75 (0·55, 1·25)	0·34 (0·24, 0·48)	0·48 (0·41, 0·57)
γ_{LLIN}	U(0, 10)	0·98 (0·60, 1·55)	0·92 (0·53, 1·62)	1·31 (0·90, 1·90)	0·83 (0·66, 1·08)	0·80 (0·51, 1·25)	1·48 (1·09, 2·01)
V_0 (6-12 weeks)	U(0, 1)	0·64 (0·37, 0·99)	0·51 (0·03, 0·96)	0·75 (0·52, 0·94)	0·60 (0·03, 0·99)	0·56 (0·10, 0·94)	0·64 (0·23, 0·90)
d_0 (6-12 weeks)	1·28 (0·11, 5·21)	0·70 (0·12, 5·62)	1·14 (0·15, 4·17)	2·29 (0·94, 4·98)	0·69 (0·13, 3·33)	1·50 (0·31, 4·88)	0·56 (0·25, 1·63)
V_{boost} (6-12 weeks)	U(0, 1)	0·31 (0·05, 0·72)	0·85 (0·28, 0·99)	0·70 (0·42, 0·94)	0·65 (0·24, 0·98)	0·92 (0·59, 0·99)	0·54 (0·30, 0·75)
d_{boost} (6-12 weeks)	1·28 (0·11, 5·21)	1·58 (0·08, 5·13)	0·60 (0·19, 2·68)	2·12 (0·48, 6·16)	1·32 (0·19, 5·01)	0·64 (0·29, 1·42)	1·97 (0·61, 5·56)
V_0 (5-17 months)	U(0, 1)	0·89 (0·66, 0·99)	0·67 (0·39, 0·89)	0·87 (0·60, 0·98)	0·92 (0·68, 0·99)		0·97 (0·87, 0·99)
d_0 (5-17 months)	1·28 (0·11, 5·21)	2·45 (0·81, 5·38)	2·64 (0·98, 6·44)	0·64 (0·37, 1·09)	0·60 (0·29, 1·21)		0·89 (0·57, 1·41)
V_{boost} (5-17 months)	U(0, 1)	0·75 (0·43, 0·99)	0·85 (0·58, 0·99)	0·27 (0·10, 0·57)	0·41 (0·13, 0·67)		0·77 (0·48, 0·97)
d_{boost} (5-17 months)	1·28 (0·11, 5·21)	0·85 (0·07, 5·07)	2·18 (0·88, 5·62)	1·03 (0·06, 4·45)	2·28 (0·37, 6·57)		2·27 (0·82, 6·26)

		Agogo	Kombewa	Kintampo	Siaya	Nanoro
Parameter	***Prior***			***Posterior***		
H_0	U(0, 100)	0·019 (0·016, 0·024)	0·092 (0·059, 0·187)	0·039 (0·032, 0·.049)	0·095 (0·075, 0·127)	0·102 (0·074, 0·147)
μ_{LN}	U(0, 100)	1·35 (1·19, 1·60)	2·62 (2·11, 3·62)	1·69 (1·51, 1·97)	2·08 (1·82, 2·42)	2·10 (1·84, 2·51)
σ_{LN}	U(0, 100)	0·92 (0·82, 1·06)	1·37 (1·18, 1·67)	1·01 (0·92, 1·15)	1·18 (1·08, 1·31)	1·16 (1·05, 1·30)
k (Γ heterogeneity)	U(0, 100)	1·05 (0·92, 1·20)	1·07 (0·97, 1·18)	1·80 (1·59, 2·05)	1·52 (1·38, 1·68)	3·53 (3·14, 3·99)
γ_{LLIN}	U(0, 10)	0·91 (0·78, 1·06)	1·06 (0·87, 1·27)	1·23 (1·07, 1·40)	1·24 (1·07, 1·41)	1·21 (0·96, 1·55)
V_0 (6-12 weeks)	U(0, 1)	0·54 (0·23, 0·89)	0·68 (0·54, 0·82)	0·19 (0·01, 0·70)	0·54 (0·36, 0·70)	0·44 (0·25, 0·62)
d_0 (6-12 weeks)	1·28 (0·11, 5·21)	0·82 (0·21, 2·81)	0·96 (0·65, 1·47)	0·50 (0·06, 4·10)	0·53 (0·31, 0·94)	0·67 (0·37, 1·41)
V_{boost} (6-12 weeks)	U(0, 1)	0·68 (0·48, 0·83)	0·61 (0·33, 0·83)	0·50 (0·23, 0·72)	0·44 (0·19, 0·68)	0·37 (0·13, 0·63)
d_{boost} (6-12 weeks)	1·28 (0·11, 5·21)	1·13 (0·59, 2·71)	0·16 (0·09, 0·30)	0·64 (0·29, 1·72)	0·28 (0·12, 2·95)	0·25 (0·09, 0·74)
V_0 (5-17 months)	U(0, 1)	0·98 (0·90, 1·00)	0·72 (0·61, 0·81)	0·90 (0·82, 0·97)	0·81 (0·73, 0·88)	0·93 (0·81, 0·99)
d_0 (5-17 months)	1·28 (0·11, 5·21)	0·53 (0·39, 0·72)	0·56 (0·38, 0·84)	0·61 (0·46, 0·83)	0·48 (0·37, 0·66)	0·71 (0·56, 0·98)
V_{boost} (5-17 months)	U(0, 1)	0·32 (0·15, 0·51)	0·54 (0·42, 0·65)	0·53 (0·42, 0·63)	0·27 (0·13, 0·43)	0·55 (0·46, 0·64)
d_{boost} (5-17 months)	1·28 (0·11, 5·21)	1·79 (0·40, 5·18)	1·33 (0·79, 2·33)	2·15 (1·25, 4·05)	0·89 (0·30, 2·65)	1·06 (0·68, 1·69)

5.3. Model validation

Figure S22 and Figure S23 show detailed model validation for Lilongwe and Nanoro, demonstrating how the age-incidence model recreates seasonality and age-incidence patterns for each of the vaccine cohorts and both age categories. Similar plots are possible for the other nine trial sites.

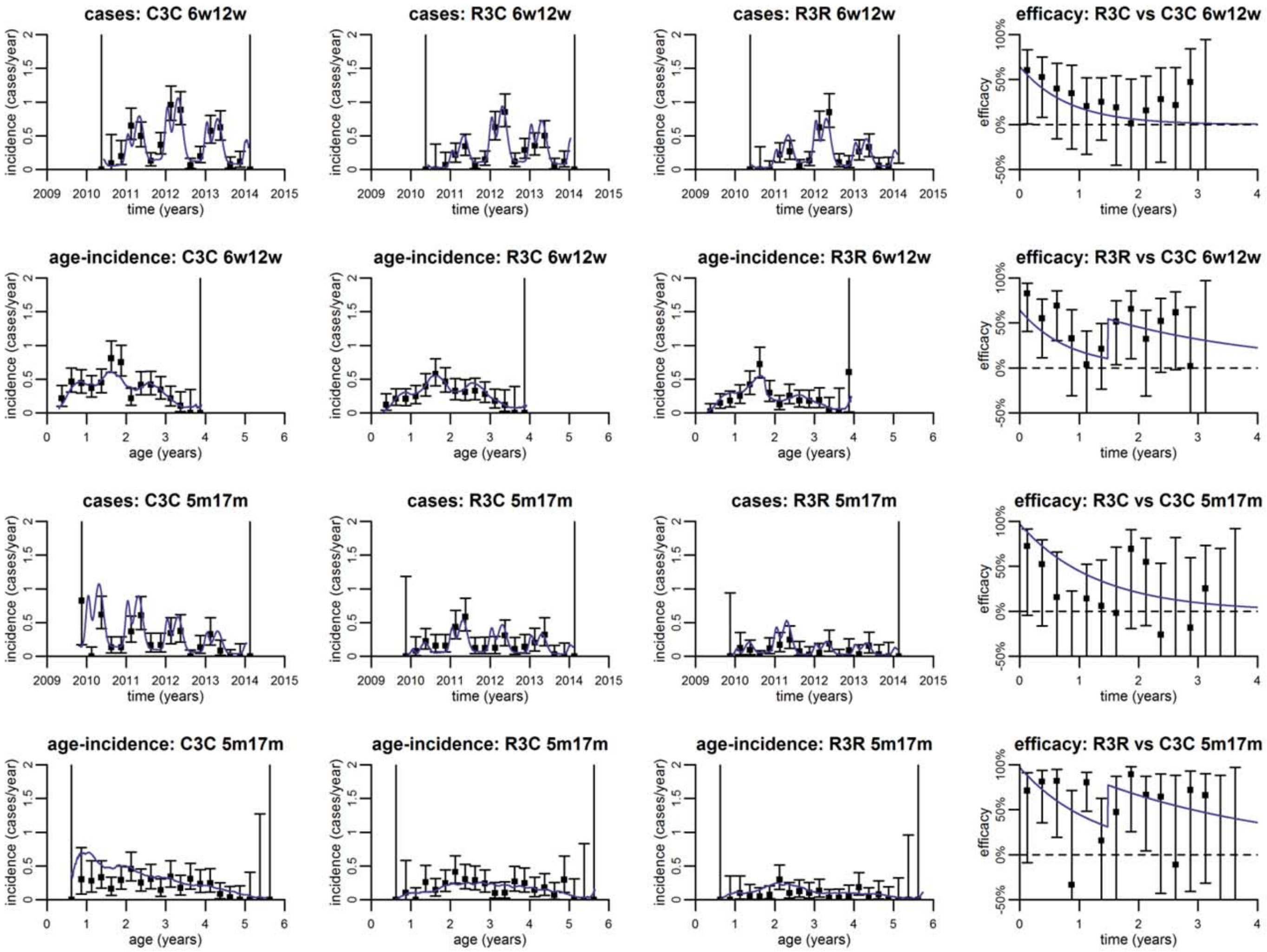

Figure S22: Validation of the age-incidence model for Lilongwe (a moderate transmission site).
An exponential vaccine efficacy profile for clinical malaria is assumed. The data are shown as cases over calendar time to present seasonal and temporal variation. Age-incidence curves are shown to present variation in incidence of clinical malaria with age. The right-hand column shows estimated efficacy against clinical malaria. Data are presented as point estimates with 95% confidence intervals. The posterior median of the age-incidence model is shown as a smooth line. 6w12w = 6-12 week age category. 5m17m = 5-17 month age category. C3C = control cohort. R3C = primary schedule of RTS,S/AS01 without a booster dose. R3R = primary schedule of RTS,S/AS01 with a booster dose.

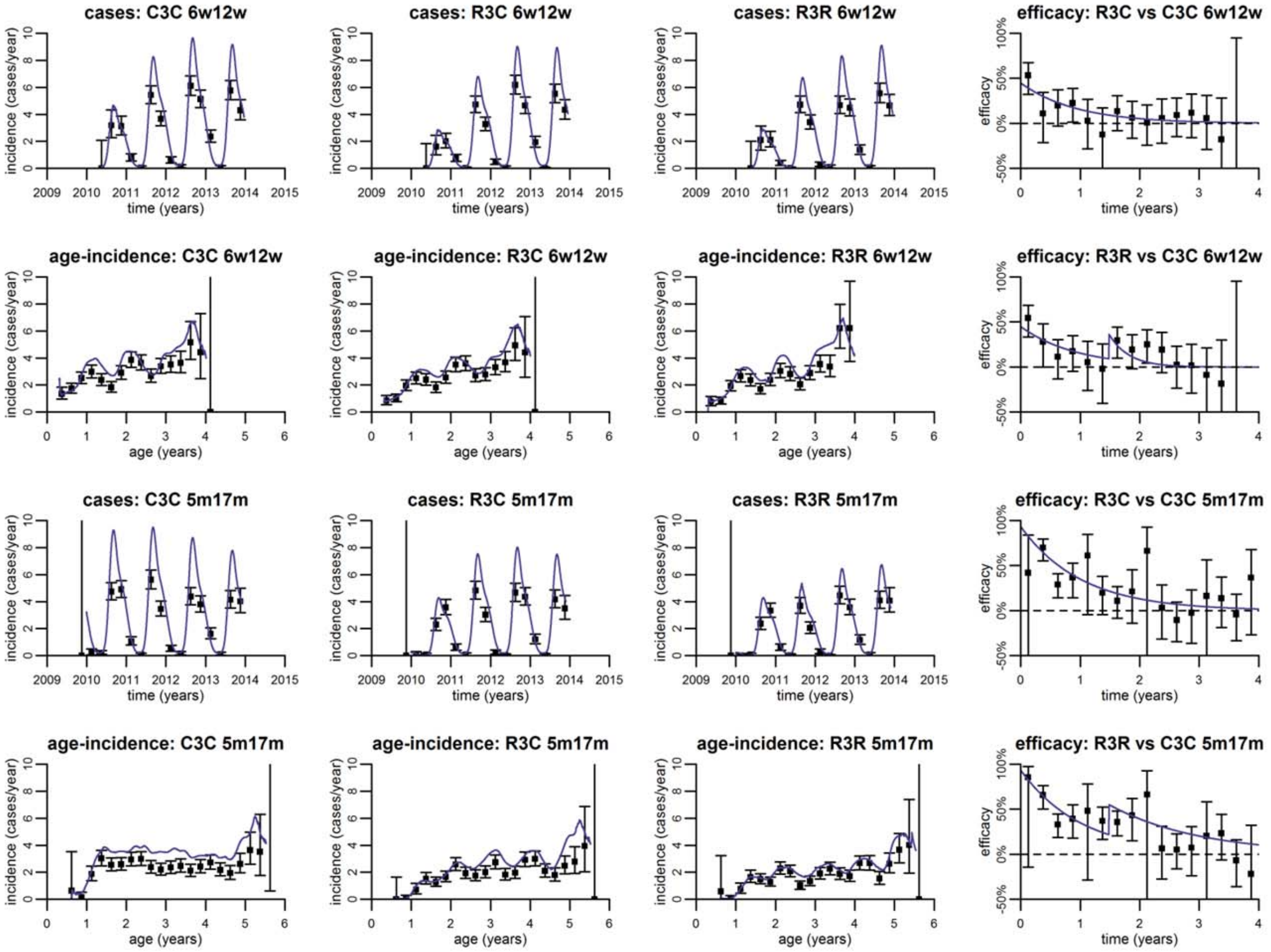

Figure S23: Validation of the age-incidence model for Nanoro (a high transmission site).

An exponential vaccine efficacy profile for clinical malaria is assumed. The data are shown as cases over calendar time to present seasonal and temporal variation. Age-incidence curves are shown to present variation in incidence of clinical malaria with age. The right-hand column shows estimated efficacy against clinical malaria. Data are presented as point estimates with 95% confidence intervals. The posterior median of the age-incidence model is shown as a smooth line. 6w12w = 6-12 week age category. 5m17m = 5-17 month age category. C3C = control cohort. R3C = primary schedule of RTS,S/AS01 without a booster dose. R3R = primary schedule of RTS,S/AS01 with a booster dose.

References

1. Smith TA. Measures of clinical malaria in field trials of interventions against *Plasmodium falciparum*. *Malaria J* 2007; **6:**53

2. Carneiro I, Roca-Feltrer A, Griffin JT, *et al*. Age-patterns of malaria vary with severity, transmission intensity and seasonality in sub-Saharan Africa: A systematic review and pooled analysis. *PLOS One* 2010; **5**(2).

3. Snow RW, Marsh K. The consequences of reducing transmission of *Plasmodium falciparum* in Africa. *Adv Parasitol* 2002; **52:**235-64.

4. Carnevale P, Frezil JL, Bosseno MF, Pont FL, Lancien J. Study of the aggressiveness of *Anopheles gambiae* A in relation to the age and sex of human subjects. *Etude de l'agressivite d'Anopheles gambiae A en fonction et de l'age et du sexe des sujets humains* 1976: 17

5. Port GR, Boreham PFL, Bryan JH. The relationship of host size to feeding by mosquitos of the *Anopheles-gambiae* giles complex (Diptera, Culicidae). *Bull Entomol Res* 1980; **70**(1): 133-44.

6. White MT, Bejon P, Olotu A, *et al*. A combined analysis of immunogenicity, antibody kinetics and vaccine efficacy from phase 2 trials of the RTS,S malaria vaccine. *BMC Medicine* 2014; 12.

7. Alonso PL, Sacarlal J, Aponte JJ, *et al*. Efficacy of the RTS,S/AS02A vaccine against *Plasmodium falciparum* infection and disease in young African children: randomised controlled trial. *Lancet* 2004; **364**(9443): 1411-20.

8. Aponte JJ, Aide P, Renom M, *et al*. Safety of the RTS,S/AS02D candidate malaria vaccine in infants living in a highly endemic area of Mozambique: a double blind randomised controlled phase I/IIb trial. *Lancet* 2007; **370**: 1543-51.

9. Kester KE, Cummings JF, Ofori-Anyinam O, *et al*. Randomized, double-blind, phase 2a trial of *Falciparum* malaria vaccines RTS,S/AS01B and RTS,S/AS02A in malaria-naive adults: Safety, efficacy, and immunologic associates of protection. *J Inf Dis* 2009; **200**(3): 337-46.

10. Polhemus ME, Remich SA, Ogutu BR, *et al*. Evaluation of RTS,S/AS02A and RTS,S/AS01B in adults in a high malaria transmission area. *PLOS One* 2009; 4(7).

11. Hu Y, Wu Q, Xu B, Zhou Z, Wang Z, Zhou Y-H. Influence of maternal antibody against hepatitis B surface antigen on active immune response to hepatitis B vaccine in infants. *Vaccine* 2008; **26**(48): 6064-7.

12. Lell B, Agnandji S, von Glasenapp I, *et al*. A randomized trial assessing the safety and immunogenicity of AS01 and AS02 adjuvanted RTS,S malaria vaccine candidates in children in Gabon. *PLOS One* 2009; 4(10).

13. Clement F, Dewar V, Van Braeckel E, *et al*. Validation of an enzyme-linked immunosorbent assay for the quantification of human IgG directed against the repeat region of the circumsporozoite protein of the parasite *Plasmodium falciparum*. *Malaria Journal* 2012; **11**.

14. Esen M, Mordmueller B, de Salazar PM, *et al*. Reduced antibody responses against *Plasmodium falciparum* vaccine candidate antigens in the presence of *Trichuris trichiura*. *Vaccine* 2012; **30**(52): 7621-4.

15. White MT, Griffin JT, Akpogheneta O, *et al*. Dynamics of the antibody response to *Plasmodium falciparum* infection in African children. *J Inf Dis* 2014; **210**(7): 1115-22.

16. Diggle PJ, Liang K-Y, Zeger SL. Oxford Statistical Science Series, 13. Analysis of longitudinal data; 1994.

17. Atchade YF, Rosenthal JS. On adaptive Markov chain Monte Carlo algorithms. *Bernoulli* 2005; **11**(5): 815-28.

18. Brooks SP, Gelman A. General methods for monitoring convergence of iterative simulations. *J Comp Graphical Stat* 1998; **7**(4): 434-55.

19. Plummer M, Best NG, Cowles K, Vines K. CODA: Convergence diagnosis and output analysis for MCMC. http://CRAN.R-project.org/Rnews; 2006.

20. Amanna IJ, Slifka MK. Mechanisms that determine plasma cell lifespan and the duration of humoral immunity. *Immunol Rev* 2010; **236**: 125-38.

21. Andraud M, Lejeune O, Musoro JZ, Ogunjimi B, Beutels P, Hens N. Living on three time scales: The dynamics of plasma cell and antibody populations iIllustrated for Hepatitis A virus. *PLOS Comp Biol* 2012; **8**(3).

22. Fraser C, Tomassini JE, Xi L, *et al*. Modeling the long-term antibody response of a human papillomavirus (HPV) virus-like particle (VLP) type 16 prophylactic vaccine. *Vaccine* 2007; **25**(21): 4324-33.

23. RTS,S Clinical Trials Partnership. First results of phase 3 trial of RTS,S/AS01 malaria vaccine in African children. *New Eng J Med* 2011; **365**(20): 1863-75.

24. RTS,S Clinical Trials Partnership. A phase 3 trial of RTS,S/AS01 malaria vaccine in African infants. *New Eng J Med* 2012; **367**(24): 2284-95.

25. Olotu A, Fegan G, Wambua J, *et al*. Four-year efficacy of RTS,S/AS01E and its interaction with malaria exposure. *New Eng J Med* 2013; **368**(12): 1111-20.

26. Guinovart C, Aponte JJ, Sacarlal J, *et al*. Insights into long-lasting protection induced by RTS,S/AS02A malaria vaccine: further results from a phase IIb trial in Mozambican children. *PLOS One* 2009; **4**(4): e5165.

27. Lievens M, Aponte JJ, Williamson J, *et al*. Statistical methodology for the evaluation of vaccine efficacy in a phase III multi-centre trial of the RTS,S/AS01 malaria vaccine in African children. *Malaria J* 2011; **10**.

28. Greco WR, Bravo G, Parsons JC. The search for synergy - a critical-review from a response-surface perspective. *Pharmacol Rev* 1995; **47**(2): 331-85.

29. Griffin JT, Hollingsworth TD, Okell LC, *et al*. Reducing *Plasmodium falciparum* malaria transmission in Africa: A model-based evaluation of intervention strategies. *PLOS Medicine* 2010; **7**(8).

30. Griffin JT. The interaction between seasonality and pulsed interventions against malaria in their effects on the reproduction number. *PLOS Comp Biol* 2015; **11**(1).

31. Patil AP, Okiro EA, Gething PW, *et al*. Defining the relationship between *Plasmodium falciparum* parasite rate and clinical disease: statistical models for disease burden estimation. *Malaria J* 2009; **8**.

32. Griffin JT, Ferguson NM, Ghani AC. Estimates of the changing age-burden of *P. falciparum* malaria disease in sub-Saharan Africa. *Nat Comms* 2014; 5.

33. Smith T, Maire N, Ross A, *et al*. Towards a comprehensive simulation model of malaria epidemiology and control. *Parasitol* 2008; **135**(13): 1507-16.

34. White M, Griffin JT, Ghani AC. The design and statistical power of treatment re-infection studies of the association between pre-erythrocytic immunity and infection with *Plasmodium falciparum*. *Malaria J* 2013; **12:** 278

35. Smith T, Ross A, maire N, *et al*. Ensemble modeling of the likely public health impact of a pre-erythrocytic malaria vaccine. *PLOS Medicine* 2012; 9(e1001157).

36. Wenger EA, Eckhoff PA. A mathematical model of the impact of present and future malaria vaccines. *Malaria J* 2013; 12.
